# Aging causes decreased resistance to multiple stresses and a failure to activate specific stress response pathways

**DOI:** 10.18632/aging.100939

**Published:** 2016-04-05

**Authors:** Dylan J. Dues, Emily K. Andrews, Claire E. Schaar, Alexis L. Bergsma, Megan M. Senchuk, Jeremy M. Van Raamsdonk

**Affiliations:** ^1^ Laboratory of Aging and Neurodegenerative Disease, Center for Neurodegenerative Science, Van Andel Research Institute, Grand Rapids, MI 49503, USA; ^2^ Department of Translational Science and Molecular Medicine, Michigan State University, Grand Rapids, MI 49503, USA; ^3^ Department of Genetics, Michigan State University, East Lansing, MI 48824, USA

**Keywords:** aging, stress resistance, C. elegans, hormesis, induced thermotolerance, heat shock response

## Abstract

In this work, we examine the relationship between stress resistance and aging. We find that resistance to multiple types of stress peaks during early adulthood and then declines with age. To dissect the underlying mechanisms, we use *C. elegans* transcriptional reporter strains that measure the activation of different stress responses including: the heat shock response, mitochondrial unfolded protein response, endoplasmic reticulum unfolded protein response, hypoxia response, SKN-1-mediated oxidative stress response, and the DAF-16-mediated stress response. We find that the decline in stress resistance with age is at least partially due to a decreased ability to activate protective mechanisms in response to stress. In contrast, we find that any baseline increase in stress caused by the advancing age is too mild to detectably upregulate any of the stress response pathways. Further exploration of how worms respond to stress with increasing age revealed that the ability to mount a hormetic response to heat stress is also lost with increasing age. Overall, this work demonstrates that resistance to all types of stress declines with age. Based on our data, we speculate that the decrease in stress resistance with advancing age results from a genetically-programmed inactivation of stress response pathways, not accumulation of damage.

## INTRODUCTION

In order to survive in a changing environment, animals must be able to respond to stresses encountered. However, activation of stress responses requires energy, and thus comes at a cost. Accordingly, animals have evolved inducible stress-responsive pathways allowing for the upregulation of stress responsive factors only upon exposure to stress. Multiple pathways have evolved to protect different compartments of the cell and to protect against different types of stresses (see Supplemental Text for full description of stress response pathways).

The heat shock response (HSR), which is also known as the cytosolic unfolded protein response, is activated in response to heat stress or other insults that disrupt protein folding in the cytoplasm. The HSR involves upregulation of various chaperones or heat shock proteins that act to restore proteostasis to the cytosol. The HSR is regulated by the heat shock factor (HSF-1) [1, 2] and can be monitored using the *Phsp-16.2::GFP* reporter strain [3, 4]. Similarly, the mitochondrial unfolded protein response (mitoUPR) [5, 6] and the endoplasmic reticulum unfolded protein response (ER-UPR) [7, 8] respond to unfolded or misfolded proteins in the mitochondria or endoplasmic reticulum, respectively. Activation of the mitoUPR can be monitored with the *Phsp-6::GFP* reporter strain [9], while *Phsp-4::GFP* worms have been used as a reporter for the ER-UPR [10].

The hypoxia response is activated under conditions of low oxygen levels (0.5%-1% oxygen) and is mediated by the HIF-1 transcription factor [11]. The promoter from the HIF-1 target gene NHR-57 has been used to generate a reporter strain for the hypoxia response (*Pnhr-57::GFP*) [12]. SKN-1 is the worm homolog of mammalian Nrf (nuclear factor erythroid related factor) proteins and has an important role in responding to oxidative stress [13]. The SKN-1-mediated oxidative stress response involves upregulation of enzymes involved in Phase II detoxification, including GST-4 and GCS-1 [14]. Reporter strains for both of these genes (*Pgst-4::GFP, Pgcs-1::GFP*) have been used to measure the SKN-1-mediated oxidative stress response [15, 16]. DAF-16 is a FOXO transcription factor that responds to various stresses. Activation of the DAF-16-mediated stress response can be monitored by examining DAF-16:GFP nuclear localization in *Pdaf-16::daf-16:GFP* worms [17]. One of the transcriptional targets of DAF-16 is the antioxidant enzyme SOD-3 whose expression can be monitored using a *Psod-3::GFP* reporter strain [18].

In this work, we use a panel of stress-responsive reporter strains to comprehensively characterize the relationship between stress resistance and aging. We show that resistance to several types of stress declines with age, but that this does not result from a detectable increase in internal stress levels. Instead, we find that the decrease in stress resistance is associated with a decreased ability to activate specific stress response pathways. In addition, we examine the role of DAF-16 and HSF-1 in activating stress responses with increasing age. We find that HSF-1 is required for induced thermotolerance to occur, while DAF-16 is needed to maintain induced thermotolerance with age.

## RESULTS

### Resistance to multiple stresses peaks during early adulthood and decreases with age

**Figure 1:**
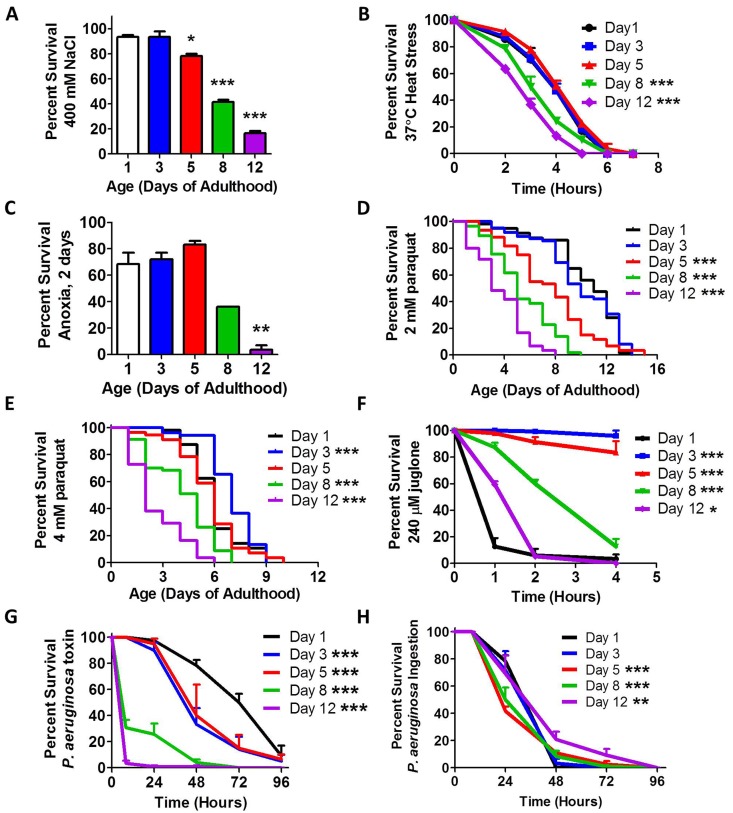
Stress resistance declines with age Synchronized populations of wild-type worms were aged to 1, 3, 5, 8 and 12 days of adulthood on plates containing 25 μM FUdR. All ages were assayed together at the same time. Multiple types of stress resistance were measured including (**A)** osmotic stress, (**B)** heat stress, (**C)** anoxia, (**D-F**) oxidative stress and (**G,H**) exposure to pathogenic bacteria. For all types of stress measured, peak resistance to stress occurred during early adulthood and declined with age. Error bars indicate SEM. *p<0.05, **p<0.01, ***p<0.001.

In order to define the relationship between stress resistance and aging, we examined resistance to osmotic stress, heat stress, anoxia, oxidative stress, and bacterial pathogenicity in wild-type worms of different ages (day 1, day 3, day 5, day 8 and day 12 of adulthood). For the osmotic stress assay, worms were transferred to plates containing 400 mM NaCl and survival was measured after 48 hours. We found that peak osmotic stress resistance occurred on day 1 and 3 of adulthood, after which there was a progressive decline (Fig 1A). Resistance to heat stress was quantified by exposing worms, which are normally grown at 20°C, to 37°C heat, and assessing survival over 8 hours. In this assay, optimal survival was exhibited by worms on days 1-5 of adulthood, followed by decreased survival in older worms (Fig 1B). Similarly, survival of worms after two days of anoxia was greatest on days 1-5 of adulthood, with decreased survival on days 8 and 12 (Fig 1C). Oxidative stress resistance was assessed using 2 mM paraquat (PQ), 4 mM PQ or 240 μM juglone. Both PQ and juglone act to increase intracellular superoxide levels through redox cycling. In each case, peak stress resistance was observed on day 3 of adulthood (Fig 1D-F). In contrast to other assays, day 1 adult worms showed the greatest sensitivity to the acute oxidative stress induced by juglone. In fact, the survival of day 1 worms was worse than 12 day old worms (See Discussion).

Finally, we assessed resistance to bacterial pathogens using *P. aeruginosa.* We performed a toxin-based assay in which killing is caused by a toxin released by the bacteria, and an ingestion-based assay where death is caused by bacterial colonization of the intestine [19]. In the toxin-based assay, we found that stress resistance was greatest at day 1 of adulthood and declined with age (Fig 1G). In the ingestion-based assay, we observed more subtle differences. We found that survival was decreased on day 5 and day 8, then increased to day 12 (Fig 1H). It is likely that the increased survival in day 12 worms results from the fact that these worms are consuming less bacteria at this age [20]. Our observation of decreased resistance to bacterial pathogen stress is consistent with a previous report in which survival on *P. aeruginosa* declined from day 3 to day 9 of adulthood [21]. The previous report shows a more dramatic decline with age, which is similar to what we observed with the toxin-based assay.

Thus, for each type of stress assayed we found that peak stress resistance occurred early in adulthood, between day 1 and day 5, followed by decreasing stress resistance with increasing age. As *C. elegans* produce almost all of their progeny before day 5 of adulthood, it would be evolutionarily advantageous to invest energy into stress resistance until this time point to allow the worm to survive until they have reproduced.

### Aging does not increase stress sufficiently to induce stress-responsive reporters

To explore the mechanism by which stress resistance decreases with age, we sought to determine if the aging process causes a measurable amount of stress, that when combined with an external stress, leads to decreased survival. For this purpose, we used fluorescent reporter strains that respond to different types of stresses and measured activation of these reporters with increasing age. We used *Phsp-16.2::GFP* worms to measure the HSR, *Phsp-6::GFP* worms to measure the mitoUPR, *Phsp-4::GFP* worms to measure the ER-UPR, *Pgst-4::GFP* and *Pgcs-1::GFP* worms to measure the SKN-1-mediated oxidative stress response, *Pnhr-57::GFP* worms to measure the HIF-1*-*mediated hypoxia response, and *Pdaf-16::daf-16::GFP* and *Psod-3::GFP* worms to measure the DAF-16-mediated stress response (DAF-16 responds to multiple types of stress) (see Supplemental Text for a summary of reporter strains used).

Before testing the activation of each reporter strain with age, we first wanted to determine which types of stress could activate each reporter and whether the reporters could be activated by multiple stresses. We exposed each reporter strain to eight different stresses: heat, cold, osmotic, anoxia, oxidative, starvation, ER, and bacterial pathogen stress. We found that many of the stress reporters were activated by multiple stresses (Fig S1-S8, Table 1). As expected, the HSR reporter *Phsp-16.2::GFP* was activated by heat stress [4], but was also induced by anoxia and mildly by a bacterial pathogen. The mitoUPR reporter *Phsp-6::GFP* was only activated by oxidative stress [22], while the ER-UPR reporter *Phsp-4::GFP* was induced by ER stress (exposure to tunicamycin) [9] and by heat [9, 23]. The *Pgst-4::GFP* and *Pgcs-1::GFP* reporters were activated by oxidative stress [15, 24] and bacterial pathogens [25]. Surprisingly, these reporters were also robustly activated by osmotic stress. The hypoxia reporter *Pnhr-57::GFP* was induced by oxidative stress [26, 27] and mildly by anoxia [28], heat, ER-stress and bacterial pathogen. The activation of the *Pdaf-16::daf-16:GFP* reporter was monitored by nuclear localization of DAF-16:GFP as DAF-16 enters the nucleus under stress. We found that heat stress [17], anoxia, oxidative stress [17], starvation [17] and bacterial pathogen stress all induced nuclear localization of DAF-16. Finally, we found that the *Psod-3::GFP* reporter was activated by heat stress, cold stress, oxidative stress [9, 29], and bacterial pathogens. Combined this indicates that multiple types of stress can activate these stress reporter strains, and using all of these reporters we have the ability to detect the presence of each type of stress tested.

**Table 1:** Stress-responsive reporter strains are activated by multiple types of stress

	Heat	Cold	Osmotic	Anoxia	Oxidative	Starvation	ER	Bacteria
*Phsp-6.2::GFP*	++	−		++			−	+
*Phsp-6::GFP*		−		−	++	−		
*Phsp-4::GFP*	++	−			−	−	++	
*Pgst-4::GFP*	−	−	++	−	++	−		++
*Pgcs-1::GFP*		−	++	−	++	−	−	+
*Pnhr-57::GFP*	+			+	++		+	+
*Pdaf-16::GFP*	Yes	No	No	Yes	Yes	Yes	No	Yes
*Psod-3::GFP*	+	+/−			+			+

Fluorescent reporter strains were exposed to a variety of stresses to determine which types of stress could activate each reporter strain. + significantly increased fluorescence in response to stress. ++ markedly increased fluorescence in response to stress (i.e. clear difference without quantification). – decreased fluorescence in response to stress. +/− increased in some regions but decreased in others. Three biological replicates were performed with similar results each time.

**Figure 2:**
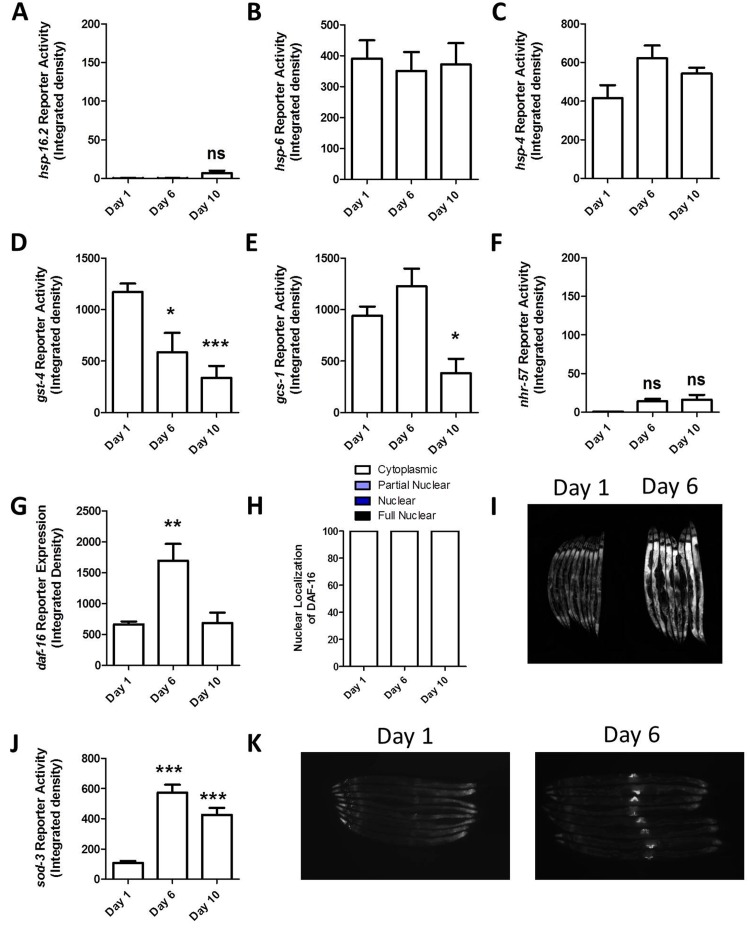
Stress-responsive reporters are not activated with increasing age To determine whether different stress response pathways are activated with increasing age, we examined fluorescence in stress-responsive reporter strains at day 1, day 6 and day 10. None of unfolded protein response reporter strains showed increased activity with age (**A-C**). The two reporters for the SKN-1 mediated oxidative stress response both showed decreased fluorescence with age (**D,E**). The hypoxia reporter also showed no increase with age (**F**). The DAF-16 reporter exhibited increased expression at day 6 (**G**) but not increased activation as measured by nuclear localization (**H,I**). Finally, the SOD-3 reporter showed increased fluorescence at both day 6 and 10 (**J**). This increase appeared to be due to a change in expression pattern with increased expression around the vulva (**K**). These results suggest that any increase in internal stress that occurs with increasing age is insufficient to activate these stress-responsive pathways and or that the stress-responsive lose their ability to respond with age. Note that the mild increase in fluorescence in *Phsp-16.2::GFP* and *Pnhr-57::GFP* worms is due to autofluorescence. Error bars indicate SEM. ns = not significant. *p<0.05, **p<0.01, ***p<0.001.

Having shown that the reporter strains can respond to stress, we next examined reporter activity with increasing age, to determine if the natural aging process elevates stress sufficiently to activate any of these different stress responses. For this purpose, we examined worms at day 1, day 6 and day 10 of adulthood. In this experiment, worms were grown without FUdR. We found that none of the unfolded protein responses (HSR, mitoUPR, ER-UPR) were induced in aged worms (Fig 2A-C, Fig S9-11). The *Pgst-4::GFP* and *Pgcs-1::GFP* reporters both showed decreased fluorescence at older ages (Fig 2D,E, Fig S12-15). The hypoxia reporter also showed no increase with age aside from autofluorescence (Fig 2F, Fig S16). We observed an increase in *Pdaf-16::daf-16:GFP* reporter expression at day 6 of adulthood, but did not observe any nuclear localization with increased age, suggesting that there is not an increase in DAF-16 activation because it remains cytoplasmic (Fig 2G-I, Fig S17). Finally, we observed increased fluorescence of the *Psod-3::GFP* reporter in aged worms (Fig 2H, Fig S18). The increase in fluorescence that we observed in *Psod-3::GFP* appeared to result from a differential expression pattern during aging: in young worms GFP expression is primarily in the head with some expression in the tail, while in older worms the brightest fluorescence occurs around the vulva (Fig 2K). Thus, it is possible that the observed increase in fluorescence levels in these worms results from a change in expression pattern rather than activation. This conclusion is consistent with our observation that DAF-16 expression is increased but not activated. In support of this conclusion, others have previously looked at *sod-3* reporter activity from day 5 to day 15 and not observed any increase in activation with advancing age [30]. Overall, the lack of induction of the stress-responsive reporter strains with age suggests that any increase in internal stress that occurs with age is too mild to activate a stress response. Alternatively, it is possible that the ability to respond to stress decreases with age.

### The ability to induce specific stress response pathways declines with age

**Figure 3:**
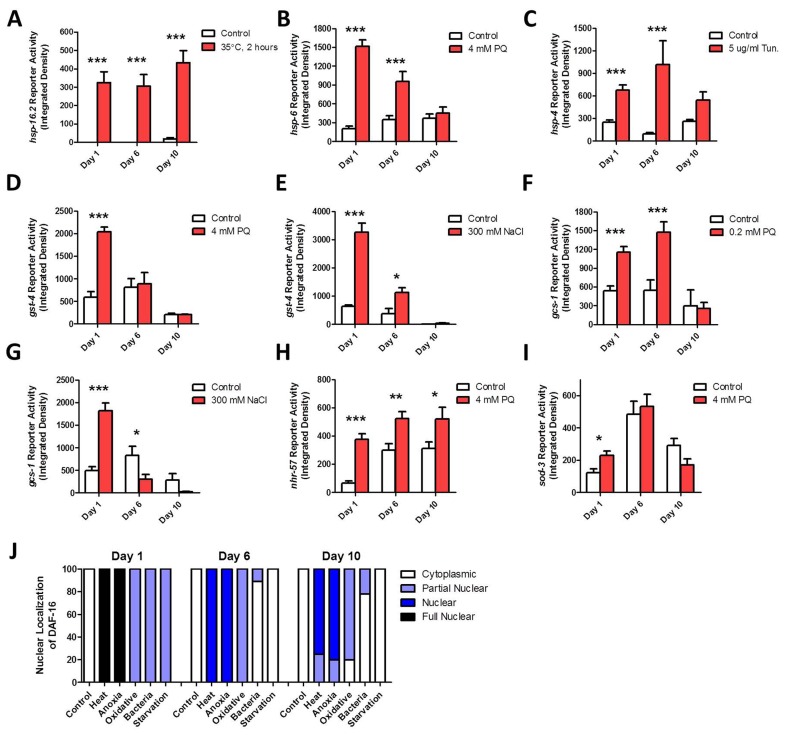
Specific stress-responsive pathways fail to respond to stress with advancing age To examine the effect of aging on the ability of specific stress-responsive pathways to become activated, we used fluorescent reporter strains and exposed worms to stress at increasing ages. The *Pgst-4::GFP* (**D,E**), *Pgcs-1::GFP* (**F,G**) and *Phsp-6::GFP* (**B**) reporter strains showed a decreased ability to respond to stress with increasing age such that by day 10 of adulthood there was no induction in response to stress. The *Psod-3::GFP* (**I**) reporter strain also failed to respond to stress on day 10 of adulthood. In contrast, the *Phsp-16.2::GFP* (**A**), *Phsp-4::GFP* (**C**), and *Pnhr-57::GFP* (**H**) reporter strains all maintained their ability to respond to stress until day 10 of adulthood. (**J**) Nuclear localization of DAF-16 could be induced by heat stress (35°C, 2 hours), anoxia (24 hours) and oxidative stress (4 mM PQ, 2 days) at all ages. Both bacterial pathogen exposure (*P. aeruginosa*) and starvation (2 days) showed a decreased ability to cause nuclear localization of DAF-16 with increasing age. Error bars indicate SEM. *p<0.05, **p<0.01, ***p<0.001.

To determine the extent to which the ability to respond to stress declines with age, we determined the optimal dose of stress for inducing each stress-responsive reporter strain (Table 2), and then exposed the reporter strains to this stress at day 1, day 6 and day 10 of adulthood. The ability to induce the HSR was maintained throughout adulthood (Fig 3A, Fig S9). In contrast, the mitoUPR showed decreased effectiveness with age, such that by day 10 of adulthood worms no longer activated the mitoUPR in response to oxidative stress (Fig 3B, Fig S10). The ER-UPR remained functional throughout adulthood, though the magnitude of induction appeared to be reduced at the oldest time point (Fig 3C, Fig S11).

**Table 2:** Optimal stress conditions to activate stress-responsive reporter strains

Reporter strain	Optimal Type of Stress	Optimal Stress Condition for Induction
*Phsp-16.2::GFP*	Heat	35°C, 2 hours + 20°C, 4 hours
*Phsp-6::GFP*	Oxidative	4 mM paraquat, 48 hours
*Phsp-4::GFP*	ER	5 ug/ml tunicamycin, 24 hours
*Pgst-4::GFP*	Osmotic	300 mM NaCl, 24 hours
*Pgcs-1::GFP*	Osmotic	300 mM NaCl, 24 hours
*Pnhr-57::GFP*	Oxidative	4 mM paraquat, 48 hours
*Pdaf-16::GFP*	Heat	35°C, 2 hours
*Psod-3::GFP*	Heat	35°C, 2 hours + 20°C, 24 hours

As both oxidative and osmotic stress were equally effective at activating the *Pgst-4::GFP* and *Phsp-4::GFP* reporter strains, we examined the ability of these reporters to respond to both types of stress with increasing age. We found that neither type of stress was able to activate either of the reporters at day 10, indicating that the SKN-1-mediated oxidative stress response is lost with age (Fig 3D-G, Fig S12-15). The hypoxia response could be induced at all three time points, though the magnitude was decreased at day 10 (Fig 3H, Fig S16) The *Psod-3::GFP* reporter could be activated at day 1 of adulthood but not at day 6 or day 10 (Fig 3I, Fig S18).

Finally, the ability of stress to induce the nuclear localization of DAF-16:GFP was tested using five different stresses. We found that heat and anoxia caused strong nuclear localization of DAF-16:GFP at all ages (Fig 3J, Fig S17). Oxidative stress caused weak nuclear localization of DAF-16 at all ages, while starvation and exposure to bacterial pathogens only induced nuclear localization of DAF-16:GFP on day 1 of adulthood (Fig 3J, Fig S17). Note that the latter result may be at least partially due to the fact that older worms eat less than younger worms [20]. This would result in less exposure to bacterial pathogen and the difference in consumption between normal eating and starvation would be much less than in early adulthood. Thus, while the reporters for the mitoUPR, SKN-1-mediated oxidative stress response and antioxidant response (*Pgst-4::GFP, Pgcs-1::GFP, Phsp-6::GFP, Psod-3:*:GFP) lose their ability to respond to stress with increasing age, the reporters for the ER-UPR, hypoxia response and DAF-16-mediated stress response (*Phsp-4::GFP, Pnhr-57::GFP, Pdaf-16::daf-16*:GFP) maintain their stress-responsiveness until at least day 10 of adulthood.

### The ability to induce a hormetic response to heat stress is lost with age

To further explore the mechanisms underlying the decline in stress resistance with increased age, we examined the ability of worms to induce a hormetic response with increasing age. It has previously been shown exposing worms to a mild heat [31-35], oxidative [33, 36], osmotic [37], hypoxic [38, 39] or cold [40] stress increases their resistance to a subsequent toxic dose of the same type of stress. In addition, for heat [31, 33] and oxidative stress [26, 41-44], exposure to a mild stress was shown to increase lifespan. To determine whether other types of stress could also induce an increase in lifespan we treated wild-type worms with mild doses of six different stresses. We found that heat stress, osmotic stress, oxidative stress and cold stress could cause an increase in lifespan (Fig S19). While we did not observe increased lifespan resulting from starvation on solid plates, a previous study found that lifespan could be increased by starving worms for 1 or 2 days in S-basal liquid media [45]. As we observed the most robust increase in lifespan with heat stress, we chose to focus on the hormetic response to heat stress in our subsequent experiments.

**Figure 4:**
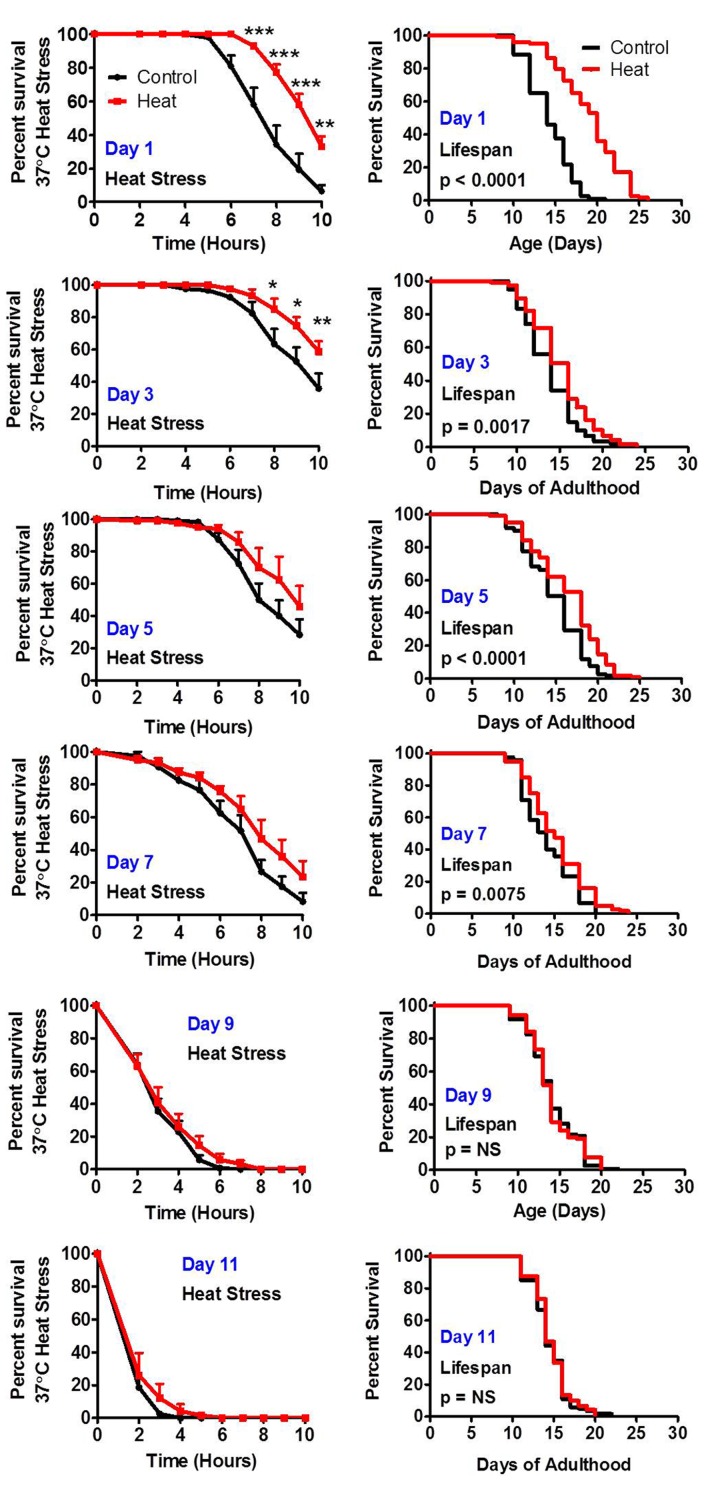
The ability to respond to stress decreases with age Worms treated with a mild 35°C heat stress exhibit increased resistance to a subsequent more severe heat stress and increased lifespan. To determine how the ability to respond to stress changes with age, worms were exposed to a mild 35°C heat stress for 2 hours at increasing ages (Day 1, Day 3, Day 5, Day 7, Day 9 and Day 11 of adulthood). The following day worms were exposed to a 37°C heat stress while a separate group was used to measure lifespan. Worms were able to respond to heat stress by increasing resistance to heat and by increasing lifespan until day 7 of adulthood inclusive.

To determine the extent to which heat stress hormesis is lost with age, we treated wild-type worms with a mild heat stress of 35°C for 2 hours at different ages. We then measured resistance to heat stress the following day and measured lifespan. We found that up until day 7 of adulthood, inclusive, that mild heat stress resulted in increased survival in the heat stress assay the following day, while at day 9 of adulthood this response was lost (Fig 4). Similarly, exposure to a mild heat stress resulted in increased lifespan up to day 7 of adulthood but not at day 9 (Fig 4). In both cases, the improvement in stress resistance or lifespan was greatest when the mild heat stress was administered on day 1 of adulthood with diminished response at older ages. Thus, although the ability to mount a hormetic response is maintained until day 7, the strength of this response is muted with increasing age. A previous study demonstrated that oxidative stress-induced hormesis using the superoxide-generator juglone is also lost with age [36].

### A single hormetic dose of heat stress improves stress resistance for multiple days

**Figure 5:**
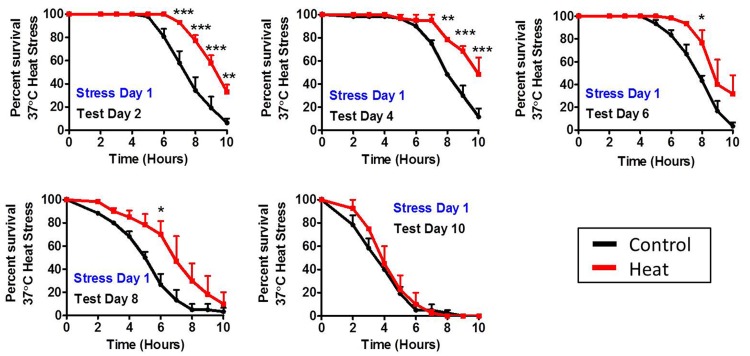
Duration of induced thermotolerance persists until day 8 of adulthood To determine the duration of induced thermotolerance, worms were exposed to a mild 35°C heat stress for 2 hours on day 1 of adulthood. Resistance to a subsequent 37°C heat stress was then tested at increasing ages (Day 2, Day 4, Day 6, Day 8 and Day 10). Worms treated with a mild heat stress maintain increased resistance to heat stress for at least 8 days. Error bars indicate SEM. *p<0.05, **p<0.01, ***p<0.001.

Since a single dose of stress was able to increase lifespan, we next sought to determine how long after exposure to a mild heat stress worms are able to maintain their induced thermotolerance. We exposed wild-type worms to a mild heat stress of 35°C for 2 hours on day 1 of adulthood and then tested resistance to heat stress at increasing ages. We found that worms tested on day 2, day 4, day 6 and day 8 of adulthood exhibited increased resistance to heat stress compared to age-matched controls (Fig 5). However, by day 10 of adulthood stress resistance was equivalent to controls.

### Activation of unfolded protein responses persist for days after stress

**Figure 6:**
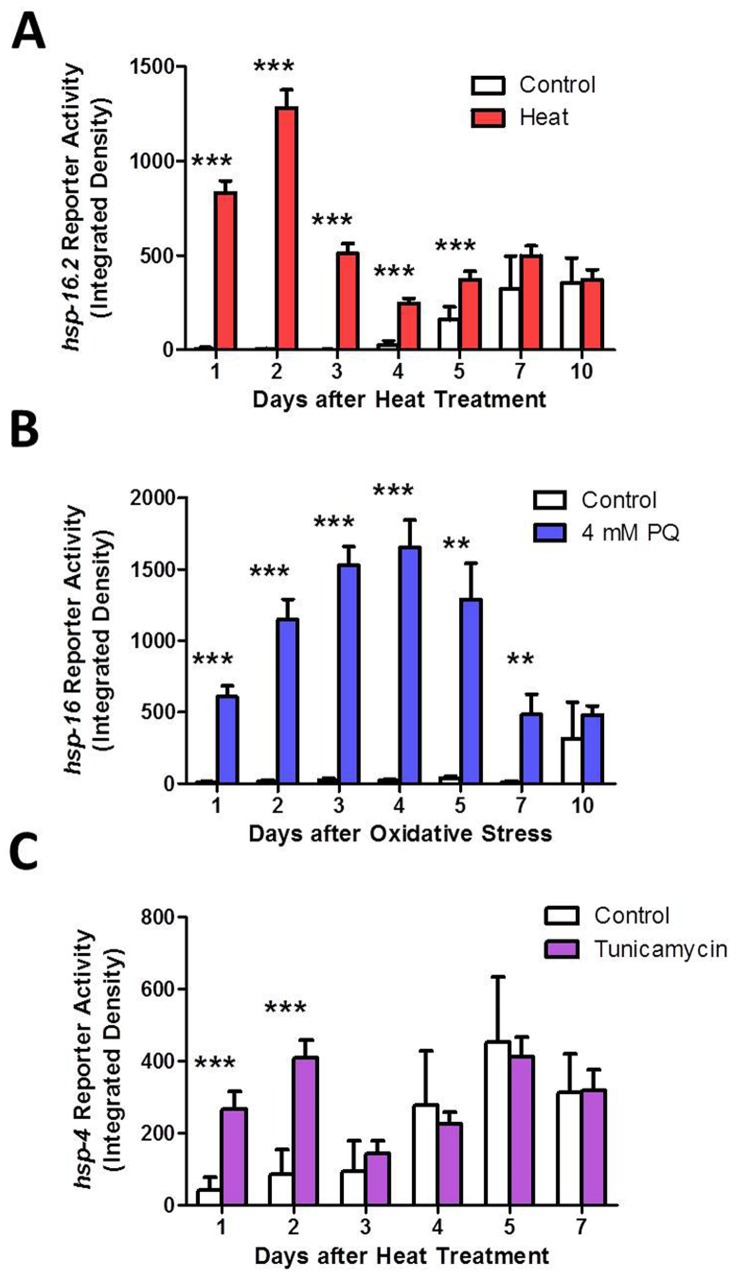
Activation of heat shock response (HSR), mitochondrial unfolded protein response (mitoUPR) and endoplasmic reticulum unfolded protein response (ER-UPR) lasts for multiple days after induction Fluorescent reporters strains were used to monitor the activation of the HSR, mitoUPR and ER-UPR following exposure to stress on day 1 of adulthood. Fluorescent reporter activity was monitored daily. (**A**) The HSR, as measured by the *Phsp-16.2::GFP* reporter strain, remains activated for at least 5 days following a mild 35°C heat stress for 2 hours. (**B)** The mitoUPR, as measured by the *Phsp-6::GFP* reporter strain, remains active for at least 7 days following exposure to 4 mM paraquat for 1 day (oxidative stress). (**C)** The ER-UPR remains active for only 2 days after exposure to 5 μg/ml tunicamycin for 1 day (ER stress). Error bars indicate SEM. *p<0.05, **p<0.01, ***p<0.001.

To explain the observation that stress resistance is increased for more than a week following a single exposure to a mild heat stress, we hypothesized that this heat stress may turn on the HSR and keep it on such that the worm would be able to avoid subsequent stresses. To test this, we exposed the HSR reporter strain *Phsp-16.2:GFP* to a mild heat stress of 35°C for 2 hours on day 1 of adulthood and then measured reporter activation until day 10 of adulthood. We found that *hsp-16.2:GFP* expression was highly induced for three days and remained elevated until day 5 of adulthood (Fig 6A, Fig S20).

To determine if the mitoUPR also remains activated for multiple days following induction, we treated the mitoUPR reporter strain *Phsp-6::GFP* with 4 mM paraquat for one day and monitored reporter activity until day 10 of adulthood. We found that the *hsp-6:GFP* expression was highly induced for 5 days following oxidative stress and was still significantly increased after 7 days (Fig 6B, Fig S21). Finally, we tested the ER-UPR by treating *Phsp-4::GFP* worms with 5 μg/ml tunicamycin for one day. We found that the ER-UPR remained activated for only two days following ER stress (Fig 6C, Fig S22). Thus, the activation of unfolded protein responses persist for multiple days following an acute stress, thereby paralleling the increase in resistance to stress.

### HSF-1 is required for induced thermotolerance, while DAF-16 is needed to maintain induced thermotolerance with age

To begin to explore the role of the stress-responsive transcription factors in induced thermotolerance, and in the activation of unfolded protein responses, we focused on DAF-16 and HSF-1 using genetic mutants. For *daf-16*, we used a deletion mutant (mu86) in which the N-termini of all of the *daf-16* transcripts are affected and is thus believed to be a null mutant [46]. For *hsf-1*, we used a point mutant for *hsf-1*, which occurs in exon 7 of 8 and results in truncation of 86 amino acids that includes the predicted transactivation domain [47, 48].

**Figure 7:**
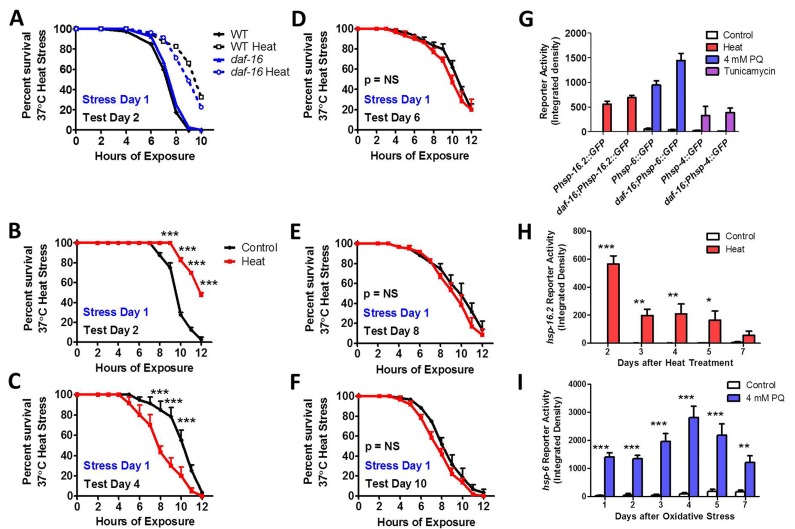
DAF-16 is required to maintain induced thermotolerance Survival under 37°C heat stress and induced thermotolerance was equivalent between *daf-16* and WT worms (**A**). Although *daf-16* worms exhibit increased survival under heat stress one day after exposure to a mild 35°C heat stress for 2 hours (**C**), the induced thermotolerance is lost less than 3 days after the heat stress (**C-F**). *daf-16* worms are able to activate the HSR, mitoUPR and ER-UPR in response to heat, oxidative stress and tunicamycin, respectively, to the same extent as WT worms at day 1 of adulthood (**G**). As in WT worms, the HSR in *daf-16* worms, as measured by *Phsp-16.2::GFP* reporter activity, remains activated for at least 5 days following a mild 35°C heat stress for 2 hours (**H**). Also, similar to WT worms, the mitoUPR in *daf-16* worms, as measured by *Phsp-6::GFP* reporter activity, remains active for at least 7 days following exposure to 4 mM paraquat for 1 day (oxidative stress). Error bars indicate SEM. *p<0.05, **p<0.01, ***p<0.001.

We found that *daf-16* worms exhibited normal sensitivity to heat stress, and induced thermotolerance of *daf-16* worms was equivalent to WT (Fig 7A). This is consistent with previous reports showing normal or mildly reduced heat stress resistance in *daf-16* worms [49-52] and an ability to increase heat stress resistance following a mild heat stress [49, 52]. However, when we examined the ability of *daf-16* worms to maintain induced thermotolerance with age, we found that the increased resistance to heat stress that resulted from exposure to a mild 35°C heat stress for 2 hours on day 1 of adulthood was lost by day 4 (Fig 7C-F).

To determine if the ability to activate unfolded protein responses is affected by disrupting DAF-16, we compared the activation of the HSR, mitoUPR and ER-UPR reporters in response to 35°C heat stress, 4 mM PQ oxidative stress and 5 μg/ml tunicamycin ER stress, respectively, to WT worms on day 1 of adulthood. We found that the activation of the *Phsp-16.2::GFP, Phsp-6::GFP* and *Phsp-4::GFP* reporters was not diminished by the *daf-16* deletion, suggesting that DAF-16 is dispensable for the HSR, mitoUPR and ER-UPR (Fig 7G, Fig S23). Since *daf-16* mutants were unable to maintain induced thermotolerance with age, we next tested the duration of activation of the HSR and mitoUPR in response to a single stress on day 1 of adulthood. As in WT worms, we found that in *daf-16* worms a single dose of stress activates the *Phsp-16.2::GFP* HSR reporter for at least 5 days (Fig 7H, Fig S24), and the *Phsp-6::GFP* mitoUPR reporter for at least 7 days (Fig 7I, Fig S25).

**Figure 8:**
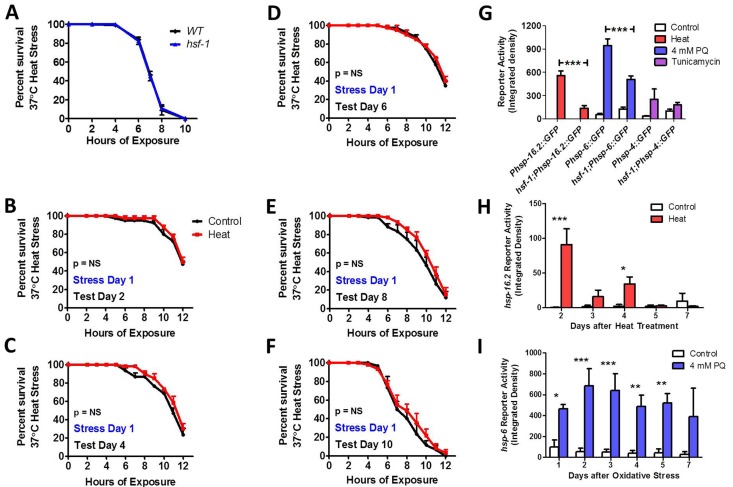
HSF-1 is required for induced thermotolerance and full activation of heat shock response and mitochondrial unfolded protein response While *hsf-1* mutant worms show equivalent 37°C heat stress survival compared to WT worms (**A**), *hsf-1* mutants failed to exhibit induced thermotolerance under heat stress after exposure to a mild 35°C heat stress for 2 hours (**B-F**). *hsf-1* mutants are able to increase *Phsp-16.2::GFP* reporter activity in response to heat stress, but the magnitude of increase is markedly less than WT (**G**). Similarly, *hsf-1* mutants show decreased induction of the *Phsp-6::GFP* mitoUPR reporter in response to oxidative stress compared to WT worms (**G**). Once activated, the duration of the HSR, as measured by *Phsp-16.2::GFP* reporter activity, is reduced in *hsf-1* worms compared to WT (**H**). Similarly, the duration of the mitoUPR in *hsf-1* worms is also decreased compared to WT worms after induction with 4 mM PQ (**I**). Error bars indicate SEM. *p<0.05, **p<0.01, ***p<0.001.

Similar to previous work [52], we find that *hsf-1* mutants are able to survive a 37°C heat stress as well as WT worms (Fig 8A), but fail to exhibit induced thermotolerance (Fig 8B-F). In examining the ability of *hsf-1* mutants to activate the HSR, mitoUPR and ER-UPR on day 1 of adulthood, we found that while *hsf-1* mutants are still able to activate the *Phsp-16.2::GFP, Phsp-6::GFP* and *Phsp-4::GFP* reporters in response to heat, oxidative and ER stress, respectively, in each case the magnitude of activation was less than in WT worms (Fig 8G, Fig S23). Decreased upregulation of the HSF-1 target gene *hsp-16.2* in response to heat stress has previously been noted [47, 53]. Finally, we tested the duration of activation of the *Phsp16.2::GFP* HSR and *Phsp-6::GFP* mitoUPR reporters in response to a single stress in *hsf-1* worms. We found that in *hsf-1* mutants *Phsp-16.2::GFP* reporter activity was increased for 4 days following heat stress (Fig 8H, Fig S26), whereas *Phsp-6::GFP* reporter activity was increased for 5 days (Fig 8I, Fig S27). Thus, in both cases the duration was less than in WT or *daf-16* worms.

## DISCUSSION

### Resistance to stress declines with age

In this work, we examine the relationship between stress resistance and aging. We assayed six different types of stress (osmotic stress, heat stress, anoxia, oxidative stress, bacterial toxin stress and bacterial ingestion stress) and found that for every stress that we tested that stress resistance declines with age (Fig S28). For each of these stresses, the peak stress resistance occurred early in adulthood between day 1 and day 5. While the pattern of decline was similar between most assays, we noted two exceptions. First, in the acute oxidative stress assay in which worms are exposed to juglone, we found that day 1 adult worms were the most sensitive. This may be due to increased permeability of the cuticle to juglone after the L4 to adult molt or possibility increased digestion of juglone as younger worms have a faster rate of pharyngeal pumping [52] and consume more bacteria [54]. The other exception was the bacterial pathogen assay, which tests resistance to intestinal colonization by *P. aeruginosa* bacteria (referred to elsewhere as the slow kill assay [19]). In this assay, we observed increased resistance in day 12 worms. Since this assay relies on ingestion of bacteria to induce toxicity, it is likely that the decreased rate of ingestion at day 12 [52, 54] contributes to their resistance.

While we were preparing our manuscript, two other groups published findings consistent with our results. Heidi Tissenbaum's group showed that resistance to both heat stress and oxidative stress decline with age [55]. In that study, worms were aged on plates containing FUdR and stress resistance was tested every 5 days. They found that resistance to heat stress and oxidative stress declined after day 5. More recently, the Morimoto lab reported that resistance to heat and oxidative stress begins to decline as early as day 1 of adulthood [21]. As this is an earlier decline than we observed or was observed by Bansal et al. [55], we wondered if the presence of FUdR could account for the differences observed, as FUdR has been shown to have effects on longevity and stress resistance for certain strains and conditions [56-59]. To test this, we compared resistance to oxidative stress in day 2 adults on NGM plates to day 2 adults on NGM plates containing 25 μM FUdR to day 1 adults. In agreement with our previous results, we found that day 2 adults are more resistant to juglone-induced oxidative stress than day 1 adults independent of the presence of FUdR (Fig S29). As the absence of FUdR does not account for the earlier decline in stress resistance observed by Labbadia et al., it may have resulted from differences in stress assay paradigms. For example, in our chronic oxidative stress assay involving paraquat, we do not observe increased sensitivity in day 1 adults (Fig 1).

Despite differences in assay conditions, all three studies clearly demonstrate that resistance to both oxidative stress and heat stress declines with age. Our study extends these previous results to show that anoxia resistance, and resistance to bacterial toxin stress also decline with increasing age.

### Aging does not activate stress response pathways

It has been proposed that accumulation of molecular damage is a primary cause of aging. For example, the free radical theory of aging suggests that oxidative damage caused by normal metabolism is a primary cause of aging [60]. Similarly, the loss of proteostasis has been proposed to be a significant driver of aging [61]. To monitor the accumulation of stress with increasing age, we used fluorescent reporter strains designed to measure a variety stress response pathways that are known to be activated by stress conditions including disruption of proteostasis or oxidative stress. Surprisingly, we did not observe activation of any of the stress-responsive reporter strains with increasing age. Further investigation revealed that this is partly due to the fact that some of the stress responsive pathways studied lose the ability to respond to stress with increased age (mitoUPR, SKN-1-mediated oxidative stress response). In contrast, the reporter strains for the HSR, ER-UPR, hypoxia response and DAF-16-mediated stress response, were still able to respond to stress at day 10 of adulthood. This indicates that any increase in internal stress levels that occurs with age is not sufficient to activate the HSR, ER-UPR, hypoxia response or DAF-16-mediated stress response. We can-not exclude the possibility that there is a mild increase in internal stress that is not sufficient to detectably increase reporter activation. Nonetheless, these results suggest that any increase in internal stress that is present is mild.

Our observation that any damage that occurs with aging is not sufficient to activate stress-response pathways is congruent with the idea that the accumulation of molecular damage is not a primary cause of aging. In other words, although increasing damage is associated with advancing age, it does not cause aging (see multiple recent reviews [62-67]). As an alternative to the widely accepted damage accumulation theories, it has been proposed that aging results from hyperfunction [68, 69]. This theory suggests that a failure to turn off signaling pathways, such as TOR (target of rapamycin), which are required for growth and differentiation during development, has detrimental effects during adulthood [70-72]. This theory provides a plausible explanation for many existing paradoxes in aging research [73], such as why larger animals between species live longer, while within a species the opposite is true [74, 75].

### Stress responsive pathways are activated by multiple types of stress

In characterizing the stress-responsive reporter strains, we examined the extent to which different types of stress could activate each stress pathway. We found that most of the stress reporter strains could be activated by multiple stresses. Heat stress was able to activate both the HSR and ER-UPR, but not the mitoUPR. It is likely that elevated heat causes protein misfolding that leads to this activation, but it is unclear why this does not affect mitochondrial proteins enough to activate the mitoUPR. A previous report also did not observe activation of the mitoUPR in response to heat stress [9]. It was surprising that osmotic stress did not activate any of the unfolded protein responses as these conditions are known to induce protein damage and aggregation [76]. It is possible that the UPR pathways do become activated at higher salt concentrations, but the salt concentration used here is sufficient to increase lifespan (Fig S19) and increased resistance to salt stress [37].

In addition to confirming that each stress responsive reporter was activated by the type of stress it was designed to detect, we also identified other stresses that could activate specific reporters. For example, we found that anoxia activated the HSR reporter *Phsp-16.2::GFP.* Perhaps most interestingly, we found that osmotic stress could activate the SKN-1-mediated oxidative stress response as measured by both the *Pgst-4::GFP* reporter and *Pgcs-1::GFP* reporter. SKN-1 has been shown to have a number of roles, including cellular stress response [13], but a role in osmotic stress resistance has not been reported. While SKN-1 was shown to be dispensable for the high osmotic stress resistance in *osm-11* mutants [77], it would be interesting to further explore the role of SKN-1 in protecting against osmotic stress.

### Role of DAF-16 and HSF-1 in unfolded protein responses

DAF-16 is a FOXO transcription factor [78, 79] that is activated in response to multiple stresses [17] and is required for the long-life and increased stress resistance of the insulin-IGF1 receptor mutant *daf-2* [31, 51, 80-82]. *daf-16* mutants have normal or mildly increased sensitivity to heat stress (Fig 7 and [49-52]), normal or increased sensitivity to oxidative stress [83-85], and a modestly decreased lifespan [80, 86, 87]. We found that although *daf-16* mutants are able to increase their thermotolerance in response to a mild heat stress as well as WT worms, the increased resistance to heat stress is rapidly lost with age. This indicates that DAF-16 is required to maintain induced thermotolerance. Surprisingly, even though heat stress resistance was lost, the duration of *Phsp-16.2::GFP* reporter activation after mild heat stress in *daf-16* worms was equivalent to WT worms. This suggests that upregulation of *hsp-16.2* is not sufficient to maintain increased thermotolerance.

The heat shock transcription factor HSF-1 is activated in response to heat stress [1, 2] and is also required for the longevity of *daf-2* worms [88]. A homozygous deletion in exon 6 of in *hsf-1(ok600)* worms results in lethality, while the *hsf-1(sy441)* point mutant used in this study results in decreased lifespan [88, 89]. Although HSF-1 is crucial in mediating the HSR, *hsf-1* mutants exhibit normal sensitivity to heat stress (Fig 8 and [52]). This suggests that initial survival of heat stress is not dependent on activation of the HSR. In contrast, HSF-1 is required for induced thermos-tolerance, which is absent in *hsf-1* mutants (Fig 8 and [52]). This may be due to the reduced ability of *hsf-1* mutants to increase expression of heat shock proteins, such as HSP-16.2 (Fig 8 and [47, 53]), in response to mild heat stress. Interestingly, we also found that *hsf-1* mutants have a decreased activation of the mitoUPR in response to oxidative stress. Nonetheless, it has been shown that *hsf-1* mutants do not have decreased survival compared to WT worms under conditions of oxidative stress [90].

### Conclusions

Stress resistance declines with age for all types of stress that we measured with maximal stress resistance occurring during early adulthood. Since early adulthood is also the peak reproductive period for the worm, this suggests the possibility that there may be a genetic program in place to maintain worm survival until reproduction has occurred – an idea which is supported by recent evidence [21]. Our data indicates that after the worm's peak reproductive period, multiple stress responsive pathways are no longer able to respond to stress, and the ability to undergo hormesis is lost. As resistance to stress has been intimately linked to longevity [91-93], understanding the molecular under-pinnings of the downregulation of stress responses with age may provide insights into improving health span.

## METHODS

### Strains and maintenance

Stress resistance assays were performed on N2 wild-type worms obtained from the *Caenorhabditis* Genetics Center. Stress responsive reporter strains were crossed into the *daf-16(mu86)* and *hsf-1(sy441)* mutant background. Wild-type males were first crossed to the fluorescent reporter strain and males from the cross were mated with *daf-16* or *hsf-1* mutants. After selfing, homozygous *daf-16* mutants were identified by PCR genotyping and homozygous *hsf-1* mutants were identified by DNA sequencing. See Supplemental Text for a full list of strains used in this study.

### Stress resistance assays

For all stress resistance assays, we used at least 3 biological replicates with a minimum of 20 worms per replicate. In piloting these studies, we observed that multiple stresses caused internal hatching of progeny, especially oxidative stress. Since this has the potential to decrease survival independently of stress resistance during the reproductive period, we maintained worms on plates containing 25 μM FUdR beginning at adulthood. This concentration of FUdR was previously shown to have minimal impact on longevity [56] and prevented internal hatching of progeny during the stress assays.

#### Osmotic stress

Resistance to osmotic stress was assessed by transferring worms to NGM plates containing 400-500 mM NaCl. Survival was monitored after 48 hours.

#### Heat stress

Heat stress resistance was measured by transferring worms to a new plate and placing the plate in a 37°C incubator. Survival was monitored hourly.

#### Anoxia

Resistance to anoxia was assessed using BD Bio-Bag Type A environmental chambers (Becton, Dikinson and Company, NJ) according to the manufacturer's instructions. Worms were transferred to 60 mm NGM plates and sealed in the bio-bags under anoxic conditions for 2 days. Worms were allowed to recover for one day before measuring survival.

#### Oxidative stress

Resistance to oxidative stress was assessed as previously described [94]. For the acute assay, worms were transferred to freshly prepared plates containing 240 or 300 μM juglone and survival was monitored hourly. For the chronic assay, worms were transferred to plates containing 2 mM paraquat and survival was monitored daily. The 2 mM paraquat plates also contained 25 μM FUdR to prevent paraquat-induced internal hatching of progeny [95].

#### Bacterial pathogen stress

We used two different assays to measure resistance to *Pseudomonas Aeruginosa.* Both assays were performed according to previously described protocols[19]. The bacterial toxin assay (referred to by Kirienko et al. as the fast kill assay) is thought to kill worms through the release of a diffusible toxin, while the bacterial ingestion assay (referred to by Kirienko et al, as the slow kill assay) is thought to act through over-colonization of the intestine.

### Hormesis for heat stress resistance and lifespan

To induce heat stress hormesis, worms were transferred to NGM plates and placed in a 35°C incubator for 2 hours to cause a mild heat stress. For stress resistance assays, worms were allowed to recover at 20°C for 24 hours before testing resistance to heat stress. For lifespan assays, NGM plates were supplemented with 25 μM FUdR. Following the mild heat stress, the lifespan plates were transferred to a 20°C incubator and survival was monitored every other day. Worms that showed internal hatching of progeny or externalization of organs were censored. To test the ability of other stresses to increase lifespan we used the following conditions: osmotic stress – 200 mM or 300 mM NaCl for 1 day; oxidative stress – 0.15 mM paraquat for 2 days or 4 mM paraquat for 1 day; cold stress – 4°C for 1 day or 10°C for 2 days; hypoxia – 6 hours or 24 hours at 0.5% oxygen; starvation – 1 day or 3 days on NGM plates.

### Exposing stress-responsive reporter strains to different types of stress

To determine which types of stresses could activate each stress reporter strain, we exposed each strain to different doses of stress and observed GFP intensity immediately following the stress and at 2, 4, 6, 24 and 48 hours following the stress. For heat stress, worms were exposed to either 30°C heat for 3 hours or 35°C heat for 2hours. For cold stress, worms were incubated at 4°C for 24 hours. For osmotic stress, worms were transferred to plates containing 300 mM NaCl. For oxidative stress, worms were transferred to plates containing 0.2 mM paraquat, 4 mM paraquat or 180 μM juglone. For anoxia, worms were placed under condition of anoxia (as described above) for 24 hours. For starvation, worms were washed three times with M9 buffer and transferred to plates containing no food. For ER stress, worms were transferred to plates containing 5 μg/ml tunicamycin. For bacterial pathogen stress worms were transferred to plates containing *P. aeruginosa* as described for the fast kill assay.

### Imaging and quantification of fluorescent reporter strains

For imaging, groups of 8 worms were picked to an unseeded NGM plate and immobilized using 20 mM sodium azide. Once immobilized worms were rapidly arranged and visualized using a Nikon SMZ1500 fluorescent microscope. Images were captured using an AVT Stingray F145B camera and VimbaViewer 1.1.2 software as previously described [96]. For quantifica-tion, images captured at the same magnification and same exposure time were combined using Adobe Photoshop into a single multi-panel image, which was opened using ImageJ software. Following subtraction of background, the multi-panel image containing all of the groups of worms to be compared was thresholded together as one image to reflect fluorescence observed by observation. In general the threshold was set above the level of autofluorescence. In the case of the *Phsp16.2::GFP* and *Pnhr-57::GFP* reporter strains, there is negligible fluorescence without induction and autofluorescence becomes brighter than uninduced reporter fluorescence at later ages. Nonetheless, when induced the reporter fluorescence is brighter than autofluorescence even at day 10. The integrated density (pixel intensity * area) of fluorescence was then measured for each worm individually.

### Nuclear localization of DAF-16:GFP

DAF-16 activation was assessed by the nuclear localization of DAF-16:GFP in the strain TJ356 ( *zIs356[Pdaf-16::daf-16a/b:GFP, rol-6(su1006)]*). The degree of nuclear localization was categorized as follows: cytoplasmic (no nuclear localization observed), partial nuclear (a small amount of DAF-:16:GFP is observed in the nucleus, but most DAF-16:GFP is cytoplasmic), nuclear (DAF-16:GFP is clearly and primarily present in the nucleus, some DAF-16:GFP is observed in the cytoplasm), and full nuclear (all DAF-16:GFP is present in the nucleus).

### Statistics

Differences between ages or times points for bar graphs was determined using one-way or two-way ANOVA followed by a Bonferroni post-test to assess differences between groups. Heat and oxidative stress assays involving multiple time points were assessed by repeated measures ANOVA. Survival plots for chronic oxidative stress assays and lifespan assays were assessed using a Log-rank test.

## SUPPLEMENTARY MATERIALS FIGURES


