# Supplementary material for: Aging causes decreased resistance to multiple stresses and a failure to activate specific stress response pathways
Source: Aging (Albany NY). 2016 Apr 5;8(4):777–95. doi: 10.18632/aging.100939 (PMC4925828; doi:10.18632/aging.100939)
Supplement: Supplementary file 1 [file aging-08-777-s001.pdf]

Supplemental Materials for:

Aging Causes Decreased Resistance to Multiple Stresses and a Failure to Activate Specific Stress Response Pathways

Dylan J. Dues¶, Emily K. Andrews¶, Claire E. Schaar¶, Alexis L. Bergsma,
Megan M. Senchuk, Jeremy M. Van Raamsdonk

Contents:

1. Supplemental Text
   - Overview of Stress Response Pathways
   - Strains Used in this Study
   - References for Supplemental Text

2. Supplemental Figures S1-S29

Supplemental Text

Overview of Stress Response Pathways

The heat shock response (HSR), which is also known as the cytosolic unfolded protein response, is the most well studied of the stress response pathways. After exposure to heat or other insults that disrupt protein folding in the cytoplasm, the heat shock factor (HSF-1), which is normally bound by an Hsp70 chaperone protein, is released, enters the nucleus, and upregulates the expression of cytosolic chaperone proteins to restore proteostasis [1, 2]. In *C. elegans,* HSP-16.2 is one of the heat shock proteins that is induced during the HSR and thus *Phsp-16.2::GFP* worms have been utilized to monitor the activation of the HSR [3, 4].

The mitochondrial unfolded protein response (mitoUPR) responds to unfolded or misfolded proteins in the mitochondria [5, 6]. A protease CLPP-1 cuts improperly folded proteins into smaller peptides which are transported out of the mitochondria by HAF-1. The exported peptides block protein import into the mitochondria, including the import of the ATFS-1 transcription factor. Since ATFS-1 has both a mitochondria targeting sequence and nuclear localization sequence, this causes ATFS-1 to enter the nucleus and upregulate mitochondrial chaperone proteins such as HSP-6. Accordingly, the activity of a *Phsp-6::GFP* reporter strain can be used to measure mitoUPR [7].

The endoplasmic reticulum unfolded protein response (ER-UPR) is involved in maintaining proteostasis in the endoplasmic reticulum [8, 9]. Unfolded proteins in the ER trigger signals to the nucleus via three transmembrane receptors: IRE-1, ATF-6 and PEK-1. Among other targets this induces the upregulation of the heat shock protein HSP-4. As such, *Phsp-4::GFP* has been used as a reporter for the ER-UPR [10].

Under conditions of low oxygen levels (0.5%-1% oxygen), worms activate a hypoxia response that is dependent on the hypoxia-inducible factor HIF-1 transcription factor [11].

When oxygen levels are high, HIF-1 is hydroxylated by EGL-9 leading to ubiquitination by the E3 ubiquitin ligase VHL-1 and degradation of HIF-1 by the proteasome [11]. However, under low oxygen conditions, there is insufficient oxygen for EGL-9 to hydroxylate HIF-1, leading to the stabilization and nuclear entry of HIF-1, where it activates gene targets that promote survival under hypoxic conditions, including the nuclear hormone receptor NHR-57 [12] . The NHR-57 promoter has been used to generate a reporter strain for the hypoxia response (*Pnhr-57::GFP*) [13].

SKN-1 is the worm homolog of mammalian Nrf (nuclear factor erythroid related factor) proteins and has an important role in responding to oxidative stress [14]. The main action of SKN-1 seems to be in upregulation of enzymes involved in Phase II detoxification, including glutathione-S-transferases, such as GST-4, and enzymes involved in the synthesis of glutathione, such as GCS-1 (gamma-glutamine cysteine synthetase) [15]. Reporter strains for both of these genes (*Pgst-4::GFP, Pgcs-1::GFP*) have been used to measure the SKN-1-mediated oxidative stress response [16, 17].

DAF-16 is a FOXO transcription factor that mediates stress responses and is also involved in the insulin-IGF1 signaling pathway. Decreasing insulin-IGF1 signaling through a mutation of the insulin-IGF1 receptor *daf-2* results in nuclear localization of DAF-16, increased lifespan and resistance to a variety of stresses [18-22]. Similarly, it has been shown using a reporter strain *Pdaf-16::daf-16:GFP* that a number of different stresses can induce the nuclear localization of DAF-16 [23]. One of the transcriptional targets of DAF-16 is the antioxidant enzyme SOD-3 whose expression can be monitored using a *Psod-3::GFP* reporter strain [24].

Strains Used in this Study

Strain Name	Genotype	Pathway	Reference
CL2070	*dvIs70[Phsp-16.2::GFP, rol-6(su1006)]*	Heat shock response/ Cytoplasmic unfolded protein response	Link et al., 1999 [3]
SJ4100	*zcIs13[Phsp-6::GFP]*	Mitochondrial unfolded protein response	Yoneda et al., 2004 [7]
SJ4005	*zcIs4[Phsp-4::GFP]*	Endoplasmic reticulum unfolded protein response	Calfon et al., 2002 [10]
CL2166	*dvIs19[Pgst-4::GFP::NLS]*	SKN-1 mediated oxidative stress response	Link and Johnson, 2002 [16]
LD1171	*idIs3[Pgcs-1::GFP, rol-6(su1006)]*	SKN-1 mediated oxidative stress response	Wang et al., 2010 [17]
ZG120	*iaIs7[Pnhr-57::GFP]*	Hypoxia response	Shen et al., 2006 [13]
TJ356	*zIs356[Pdaf-16::daf-16a/b::GFP, rol-6(su1006)]*	DAF-16 mediated stress response	Henderson and Johnson, 2005 [23]
CF1553	*muIs84[Psod-3::GFP, rol-6(su1006)]*	DAF-16 mediated stress response	Libina et al., 2003 [24]
JVR397	*daf-16(mu86);dvIs70[Phsp-16.2::GFP, rol-6(su1006)]*	Heat shock response/ Cytoplasmic unfolded protein response	This study
JVR395	*daf-16(mu86); zcIs13[Phsp-6::GFP]*	Mitochondrial unfolded protein response	This study
JVR396	*daf-16(mu86); zcIs4[Phsp-4::GFP]*	Endoplasmic reticulum unfolded protein response	This study
JVR399	*hsf-1(sy441);dvIs70[Phsp-16.2::GFP, rol-6(su1006)]*	Heat shock response/ Cytoplasmic unfolded protein response	This study
JVR398	*hsf-1(sy441); zcIs13[Phsp-6::GFP]*	Mitochondrial unfolded protein response	This study
JVR400	*hsf-1(sy441); zcIs4[Phsp-4::GFP]*	Endoplasmic reticulum unfolded protein response	This study

References for Supplemental Text

1. Cotto JJ and Morimoto RI. Stress-induced activation of the heat-shock response: cell and molecular biology of heat-shock factors. Biochemical Society symposium. 1999; 64:105-118.
2. Vabulas RM, Raychaudhuri S, Hayer-Hartl M and Hartl FU. Protein folding in the cytoplasm and the heat shock response. Cold Spring Harbor perspectives in biology. 2010; 2(12):a004390.
3. Link CD, Cypser JR, Johnson CJ and Johnson TE. Direct observation of stress response in Caenorhabditis elegans using a reporter transgene. Cell Stress Chaperones. 1999; 4(4):235-242.
4. Rea SL, Wu D, Cypser JR, Vaupel JW and Johnson TE. A stress-sensitive reporter predicts longevity in isogenic populations of Caenorhabditis elegans. Nat Genet. 2005; 37(8):894-898.
5. Haynes CM and Ron D. The mitochondrial UPR - protecting organelle protein homeostasis. Journal of cell science. 2010; 123(Pt 22):3849-3855.
6. Jovaisaite V, Mouchiroud L and Auwerx J. The mitochondrial unfolded protein response, a conserved stress response pathway with implications in health and disease. The Journal of experimental biology. 2014; 217(Pt 1):137-143.
7. Yoneda T, Benedetti C, Urano F, Clark SG, Harding HP and Ron D. Compartment-specific perturbation of protein handling activates genes encoding mitochondrial chaperones. Journal of cell science. 2004; 117(Pt 18):4055-4066.
8. Mori K. Signalling pathways in the unfolded protein response: development from yeast to mammals. Journal of biochemistry. 2009; 146(6):743-750.
9. Walter P and Ron D. The unfolded protein response: from stress pathway to homeostatic regulation. Science. 2011; 334(6059):1081-1086.
10. Calfon M, Zeng H, Urano F, Till JH, Hubbard SR, Harding HP, Clark SG and Ron D. IRE1 couples endoplasmic reticulum load to secretory capacity by processing the XBP-1 mRNA. Nature. 2002; 415(6867):92-96.
11. Powell-Coffman JA. Hypoxia signaling and resistance in C. elegans. Trends in endocrinology and metabolism: TEM. 2010; 21(7):435-440.
12. Shen C, Nettleton D, Jiang M, Kim SK and Powell-Coffman JA. Roles of the HIF-1 hypoxia-inducible factor during hypoxia response in Caenorhabditis elegans. J Biol Chem. 2005; 280(21):20580-20588.
13. Shen C, Shao Z and Powell-Coffman JA. The Caenorhabditis elegans rhy-1 gene inhibits HIF-1 hypoxia-inducible factor activity in a negative feedback loop that does not include vhl-1. Genetics. 2006; 174(3):1205-1214.
14. Blackwell TK, Steinbaugh MJ, Hourihan JM, Ewald CY and Isik M. SKN-1/Nrf, stress responses, and aging in Caenorhabditis elegans. Free Radic Biol Med. 2015.
15. Tullet JM, Hertweck M, An JH, Baker J, Hwang JY, Liu S, Oliveira RP, Baumeister R and Blackwell TK. Direct inhibition of the longevity-promoting factor SKN-1 by insulin-like signaling in C. elegans. Cell. 2008; 132(6):1025-1038.
16. Link CD and Johnson CJ. Reporter transgenes for study of oxidant stress in Caenorhabditis elegans. Methods in enzymology. 2002; 353:497-505.

17. Wang J, Robida-Stubbs S, Tullet JM, Rual JF, Vidal M and Blackwell TK. RNAi screening implicates a SKN-1-dependent transcriptional response in stress resistance and longevity deriving from translation inhibition. PLoS Genet. 2010; 6(8).
18. Kenyon C, Chang J, Gensch E, Rudner A and Tabtiang R. A C. elegans mutant that lives twice as long as wild type. Nature. 1993; 366(6454):461-464.
19. Lithgow GJ, White TM, Melov S and Johnson TE. Thermotolerance and extended life-span conferred by single-gene mutations and induced by thermal stress. Proc Natl Acad Sci U S A. 1995; 92(16):7540-7544.
20. Warnhoff K, Murphy JT, Kumar S, Schneider DL, Peterson M, Hsu S, Guthrie J, Robertson JD and Kornfeld K. The DAF-16 FOXO transcription factor regulates natc-1 to modulate stress resistance in Caenorhabditis elegans, linking insulin/IGF-1 signaling to protein N-terminal acetylation. PLoS Genet. 2014; 10(10):e1004703.
21. Lamitina ST and Strange K. Transcriptional targets of DAF-16 insulin signaling pathway protect C. elegans from extreme hypertonic stress. Am J Physiol Cell Physiol. 2005; 288(2):C467-474.
22. Scott BA, Avidan MS and Crowder CM. Regulation of hypoxic death in C. elegans by the insulin/IGF receptor homolog DAF-2. Science. 2002; 296(5577):2388-2391.
23. Henderson ST and Johnson TE. daf-16 integrates developmental and environmental inputs to mediate aging in the nematode Caenorhabditis elegans. Curr Biol. 2001; 11(24):1975-1980.
24. Libina N, Berman JR and Kenyon C. Tissue-specific activities of C. elegans DAF-16 in the regulation of lifespan. Cell. 2003; 115(4):489-502.

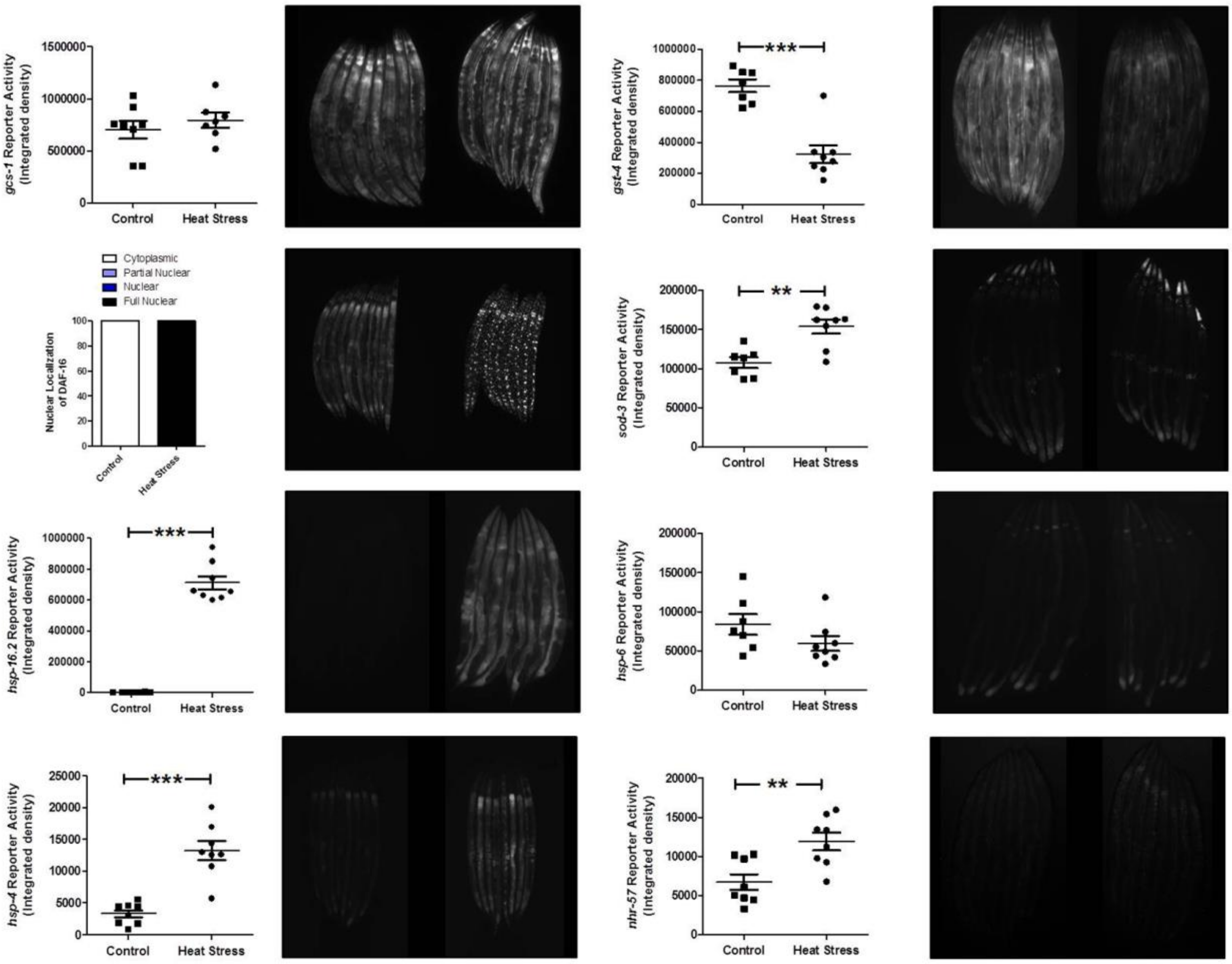

Supplemental Figure S1. Activation of stress-responsive reporters by heat stress. Day 1 adult worms were exposed to 35ºC heat stress for 2 hours and imaged between 0 and 4 hours thereafter when response was optimal. We found that heat stress induced nuclear localization of the *Pdaf-16::daf-16:GFP* reporter and increased expression of the *Psod-3::GFP, Phsp-16.2::GFP* and *Pnhr-57::GFP* reporters. While adult *Phsp-4::GFP* worms did not respond to 35ºC heat stress (not shown), we found that L4 worms exposed to 30ºC heat stress for 3 hours exhibited increased expression of GFP from the *hsp-4* promoter. **p<0.01, ***p<0.001.

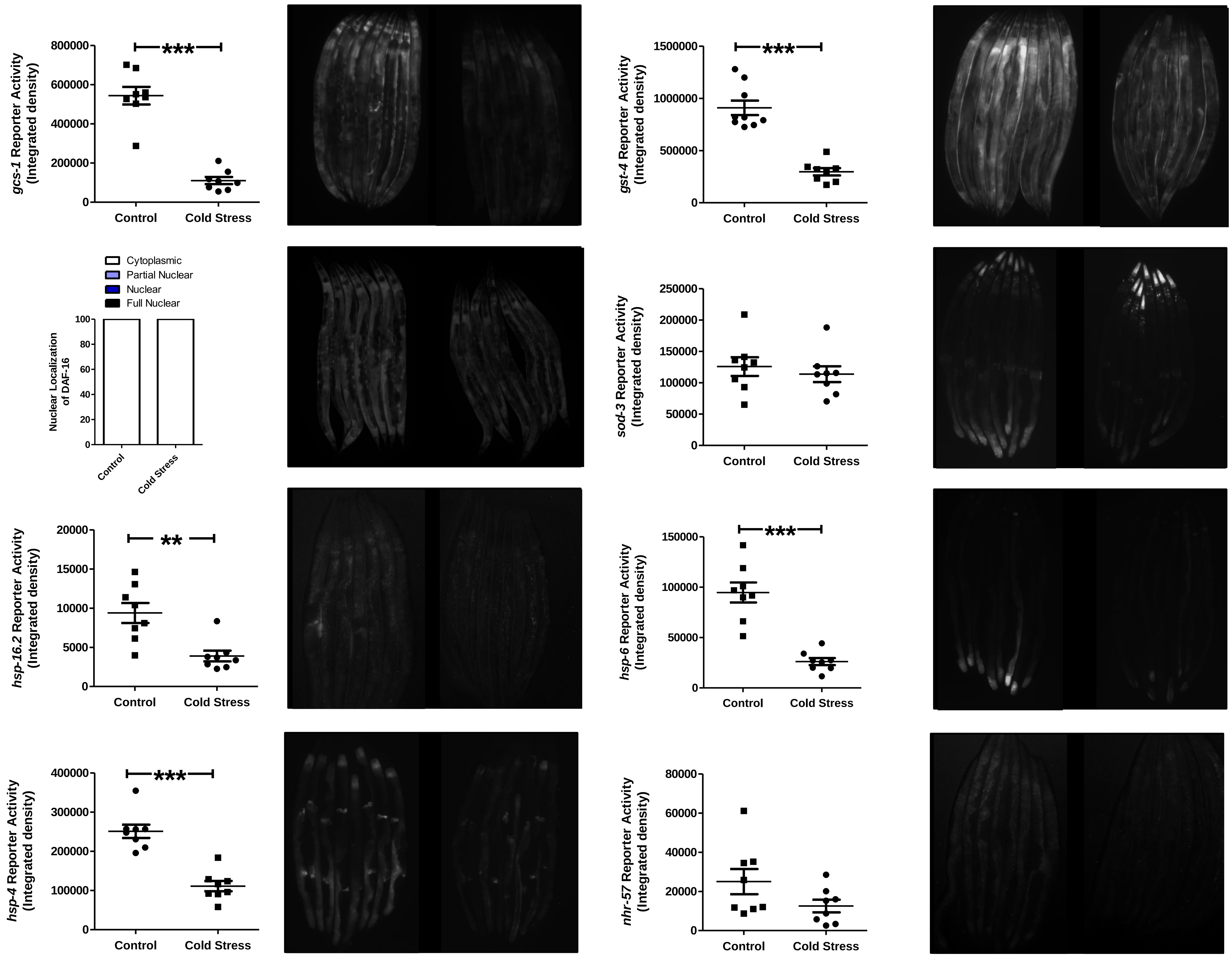

Supplemental Figure S2. Activation of stress-responsive reporters by cold stress. Day 1 adult worms were exposed to 4°C cold stress for 24 hours and imaged between 0 and 24 hours thereafter when response was optimal. In general, we found that exposing worms to cold resulted in decreased reporter activity. This response likely results from a non-specific slowing of protein synthesis. The *Psod-3:GFP* reporter strain exhibited a re-localization of GFP expression with diminished GFP levels in the tail and increased GFP in the head. Exposure to cold or reheating to 20°C did not induce the nuclear localization of DAF-16. **p<0.01, ***p<0.001.

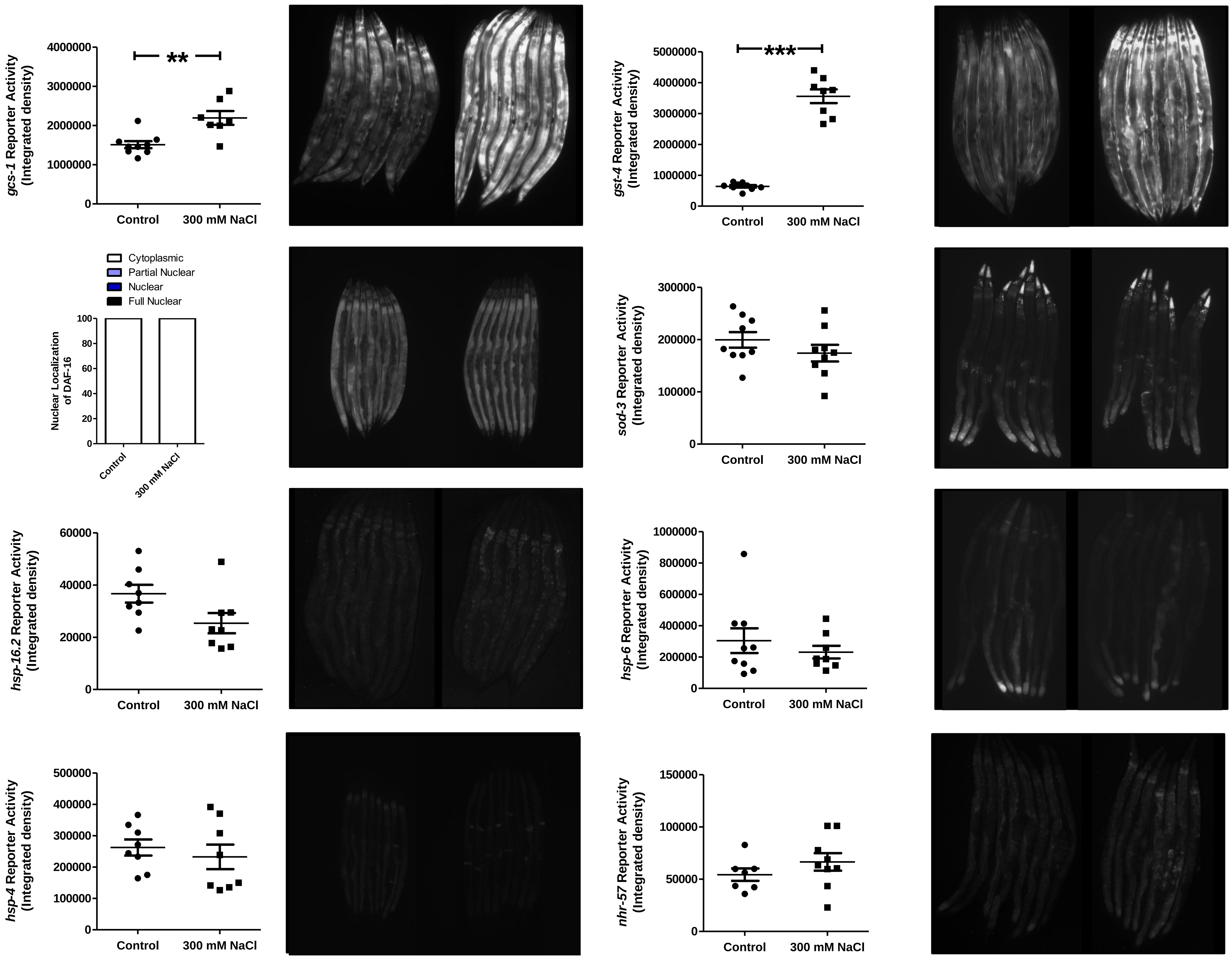

Supplemental Figure S3. Activation of stress-responsive reporters by osmotic stress. Day 1 adult worms were exposed to 300 mM NaCl for 24 hours and imaged. While most of the reporter strains did not respond to osmotic stress, both of the *skn-1* target reporter strains, *Pgst-4::GFP* and *Pgcs-1::GFP*, were activated by under these conditions. **p<0.01, ***p<0.001.

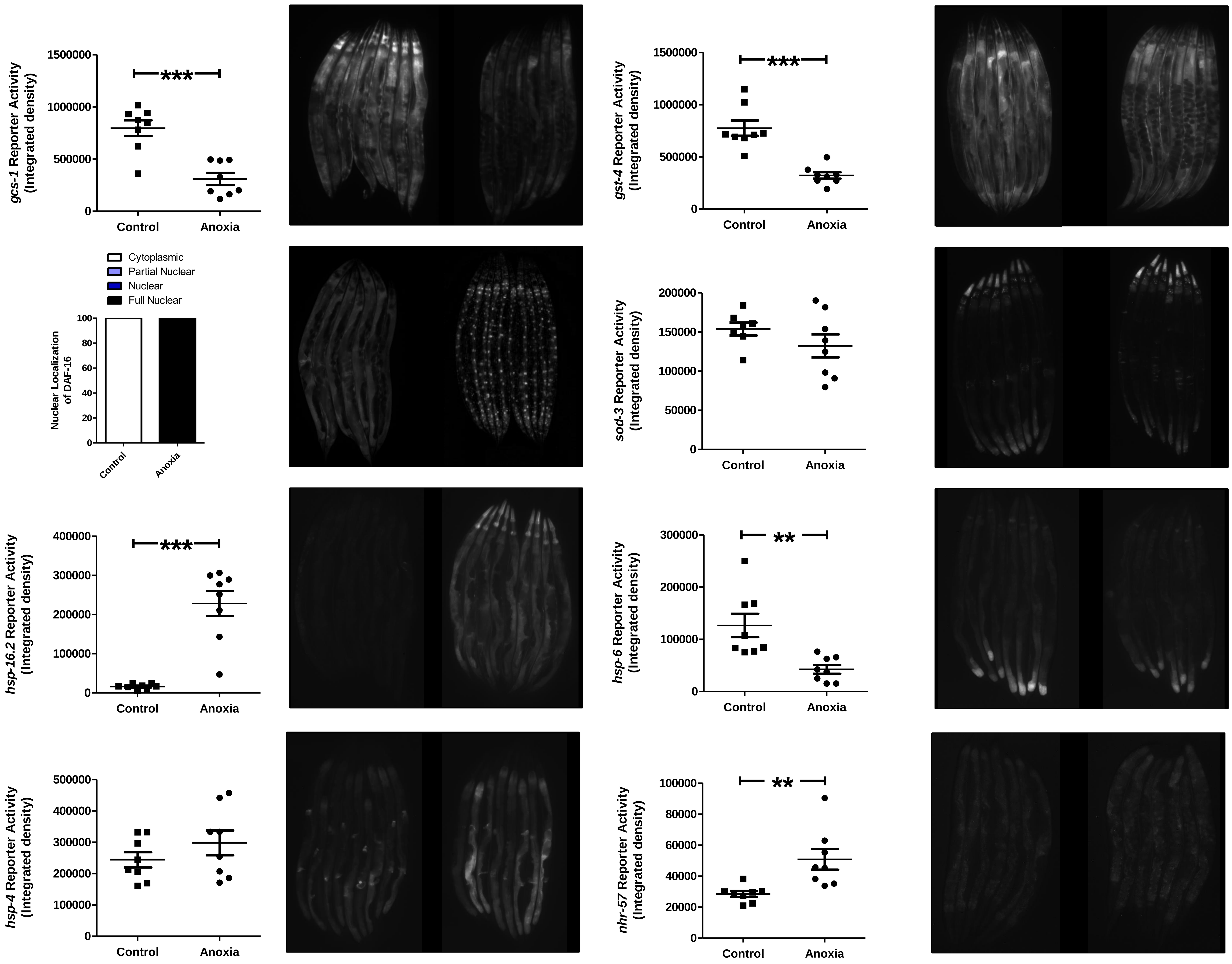

Supplemental Figure S4. Activation of stress-responsive reporters by anoxia. Day 1 adult worms were exposed to complete anoxia for 24 hours and imaged. While most of the reporter strains did not respond to anoxia or showed decreased expression, we found that the HSR reporter *Phsp-16.2:GFP* was robustly activated by anoxia. In addition, we observed mild activation of the hypoxia reporter *Pnhr-57::GFP.* **p<0.01, ***p<0.001.

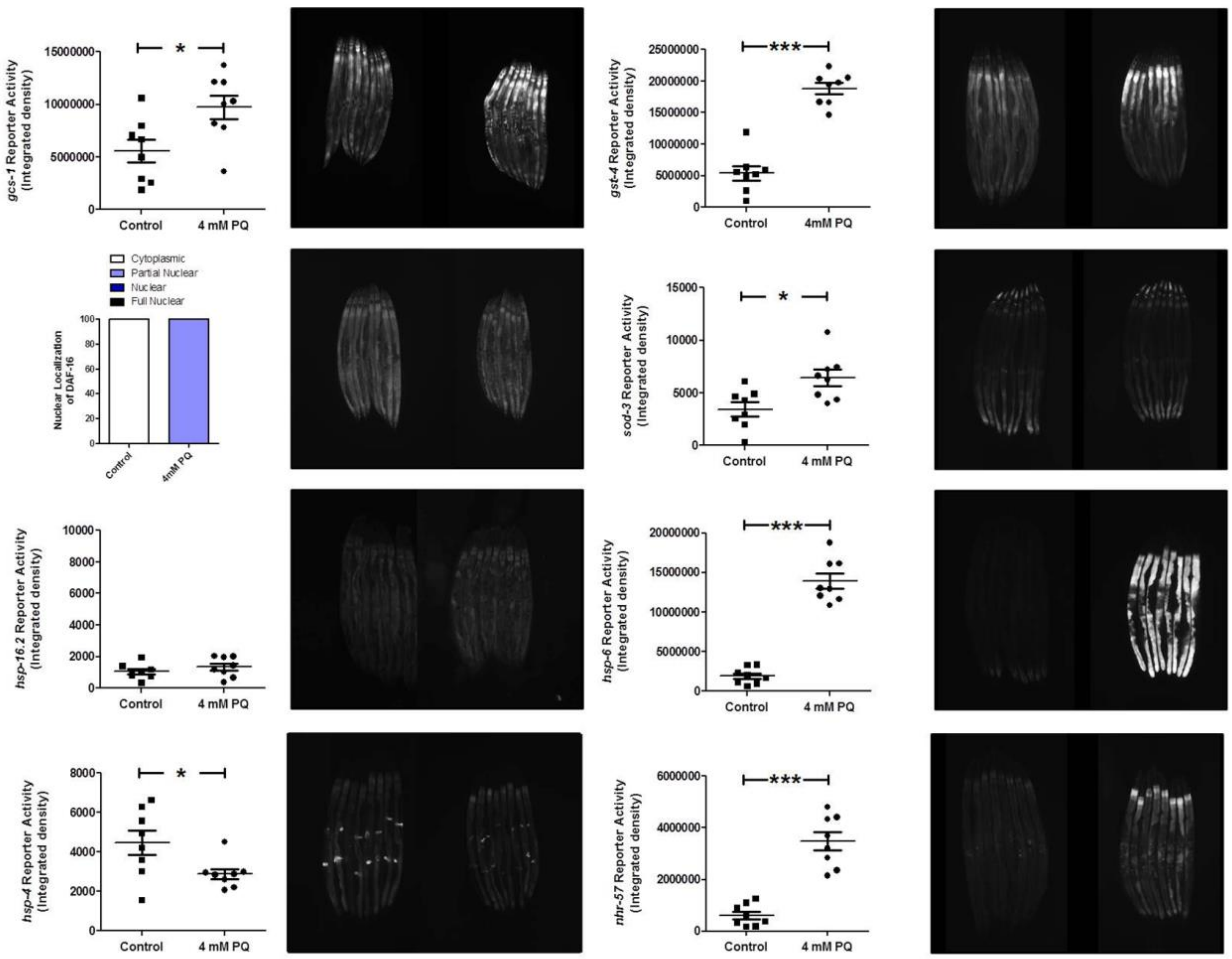

Supplemental Figure S5. Activation of stress-responsive reporters by oxidative stress. Day 1 adult worms were exposed to 4 mM paraquat for 48 hours and imaged. Multiple stress responsive reporter strains were activated by oxidative stress including *Pgst-4::GFP, Pgcs-1::GFP, Pdaf-16::daf-16:GFP* (partial nuclear localization), *Phsp-6::GFP* and *Pnhr-57::GFP.* *p<0.05, **p<0.01, ***p<0.001.

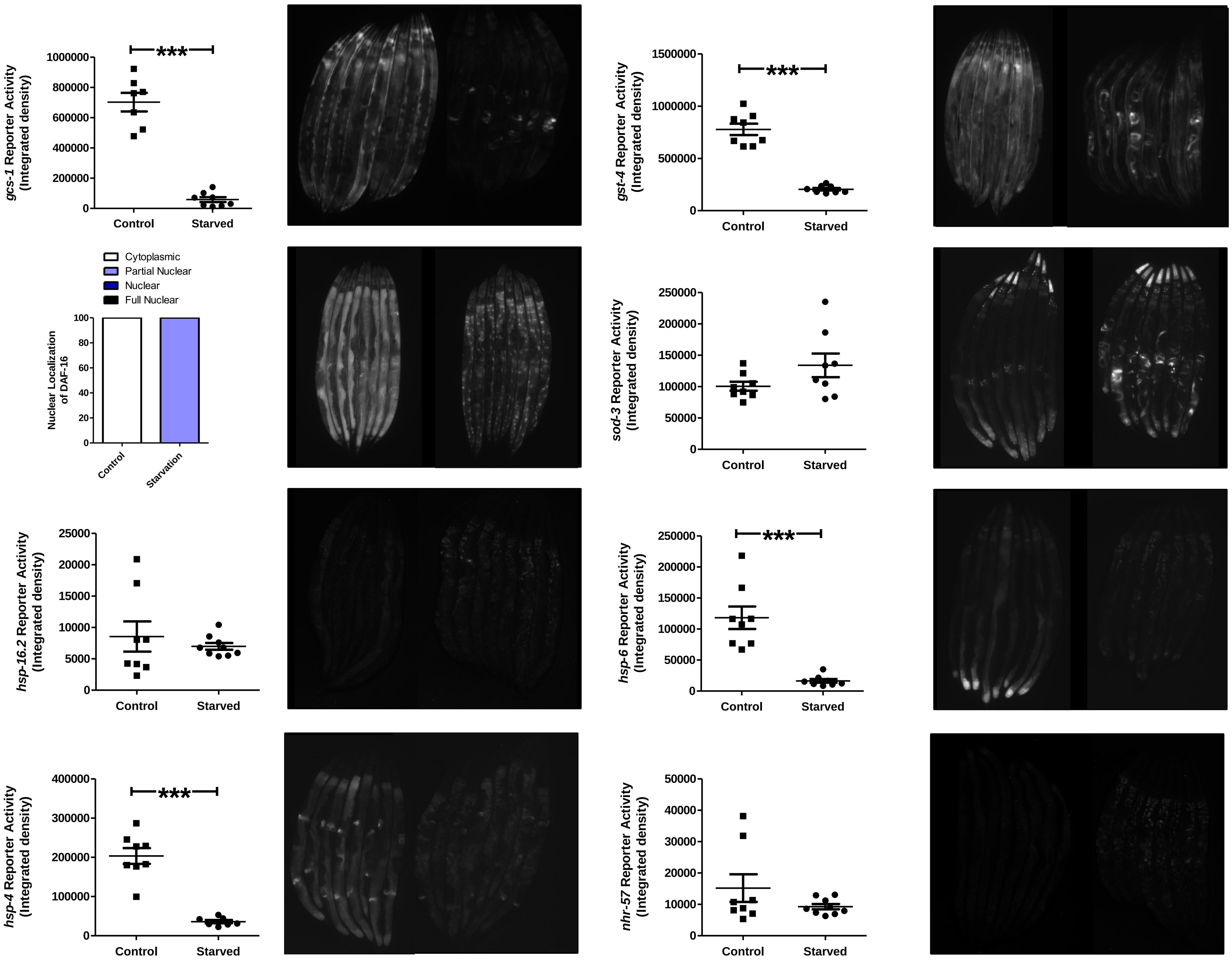

Supplemental Figure S6. Activation of stress-responsive reporters by starvation. Day 1 adult worms were washed three times with M9 buffer and transferred to an NGM plate containing no food. Worms were imaged after 1-2 days. We found that multiple reporters showed decreased expression under starvation conditions including *Pgst-4::GFP, Pgcs-1::GFP, Phsp-6::GFP* and *Phsp-4::GFP.* This is likely due to decreased levels of protein synthesis. In contrast, we found that starvation induced the nuclear localization of DAF-16 and increased expression of *Psod-3::GFP.* ***p<0.001.

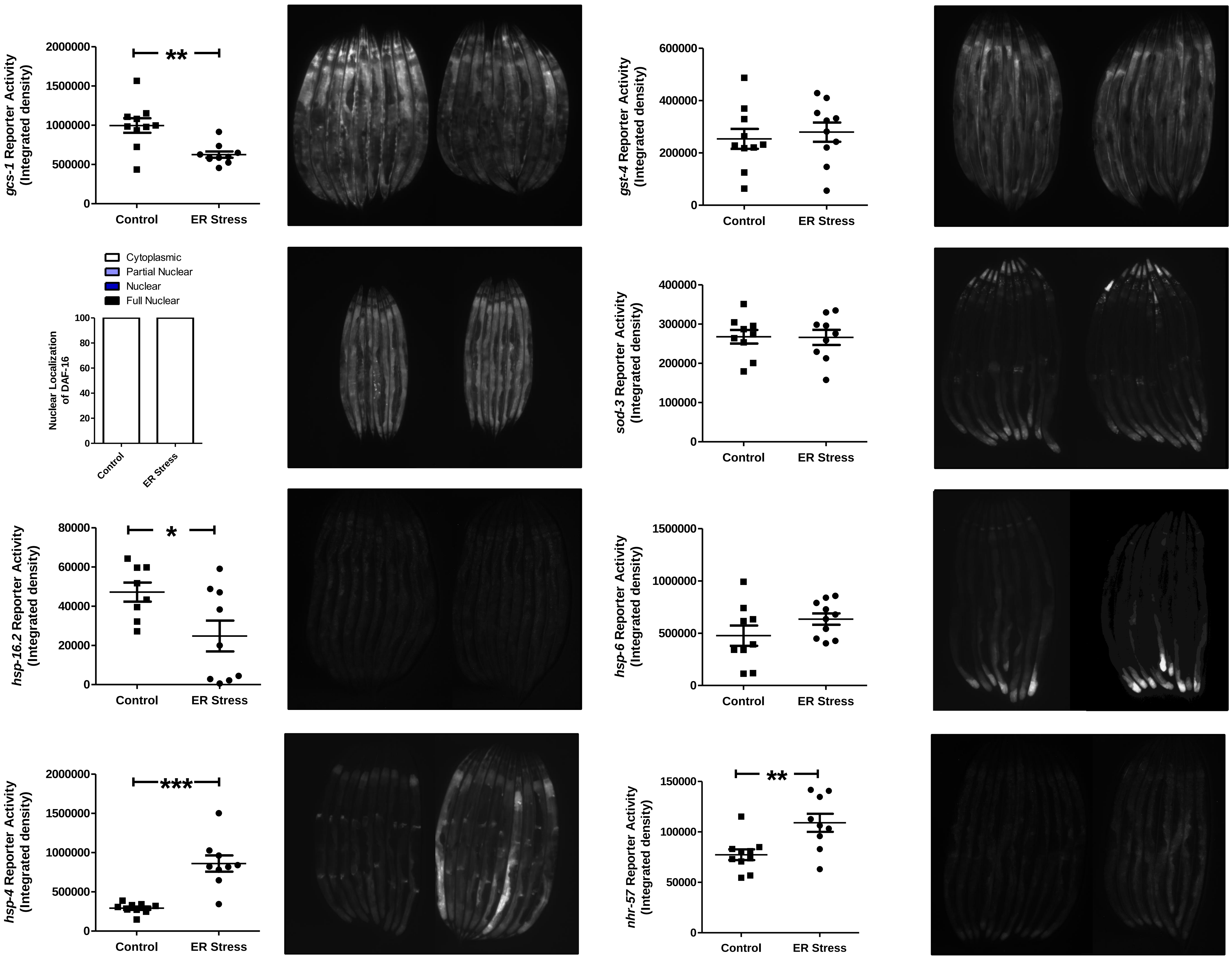

Supplemental Figure S7. Activation of stress-responsive reporters by ER stress. Day 1 adult worms were exposed to 5 µg/ml tunicamycin for 1 day and then imaged. As anticipated ER stress induced the ER-UPR reporter strain *Phsp-4::GFP.* We also observed a very mild activation of *Pnhr-57::GFP.* **p<0.01, ***p<0.001.

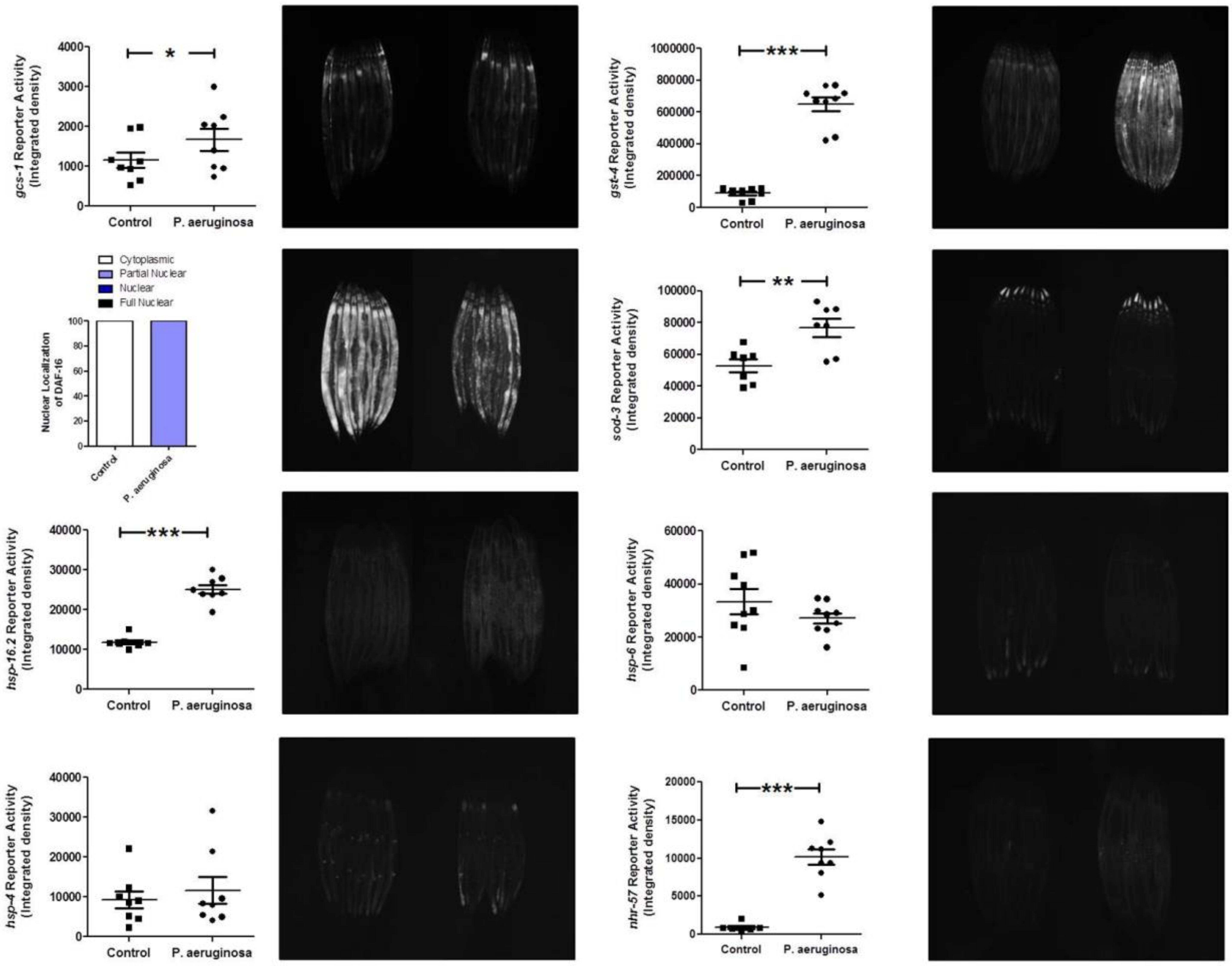

Supplemental Figure S8. Activation of stress-responsive reporters by a bacterial pathogen. Day 1 adult worms were exposed to *Pseudomonas aeruginosa* for 4-24 hours and imaged when activation was greatest. Multiple stress responsive reporter strains were activated by bacterial pathogen stress including *Pgst-4::GFP, Pdaf-16::daf-16:GFP* (nuclear localization), *Psod-3::GFP, Phsp-16.2::GFP* and *Pnhr-57::GFP.* The peak response of the *Pgst-4::GFP* reporter occurred at 24 hours. The *Pgcs-1::GFP* reporter strain showed decreased survival such that imaging had to be performed at 4 hours. **p<0.01, ***p<0.001.

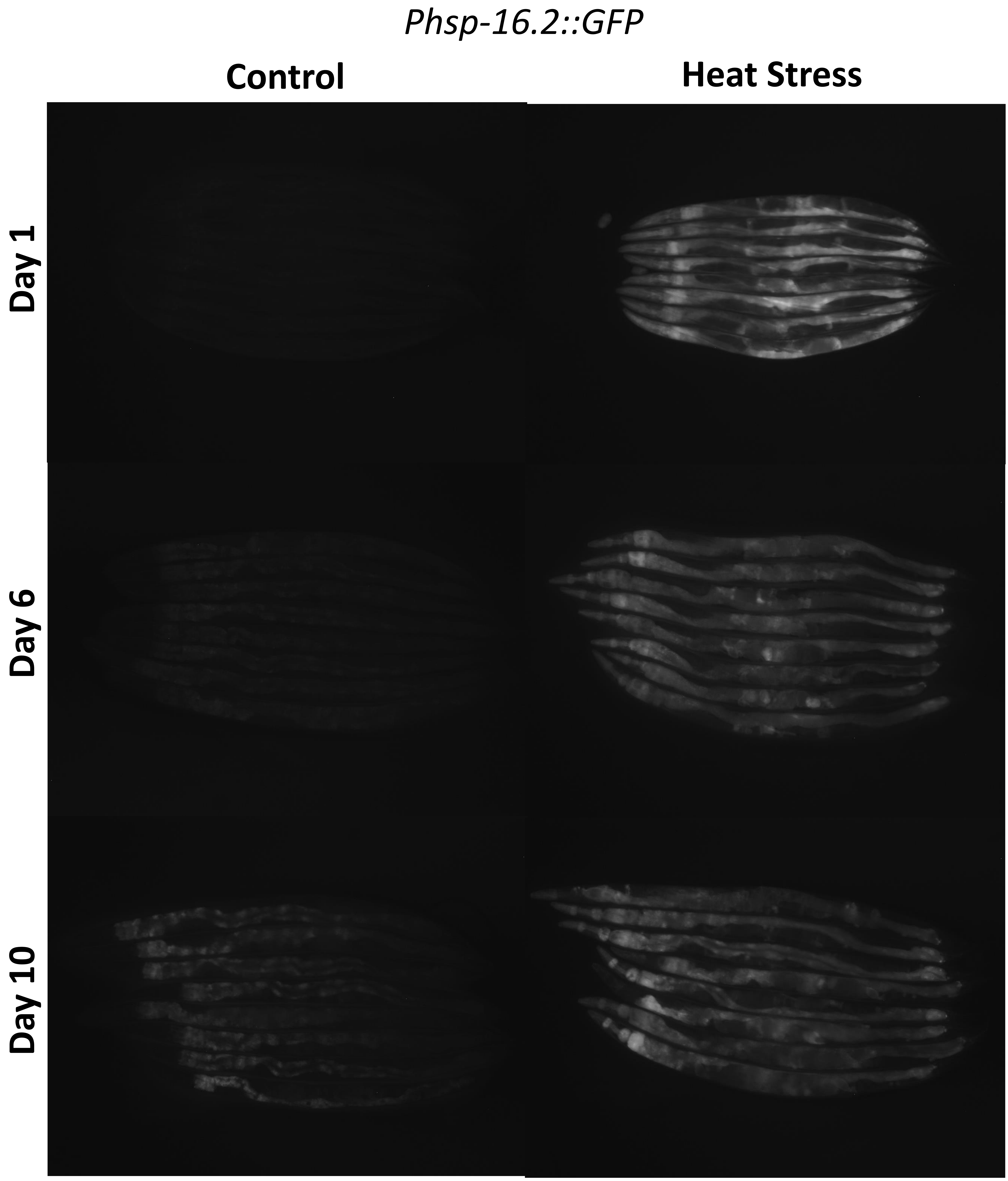

Supplemental Figure S9. Ability to activate heat shock response (HSR) is maintained throughout adulthood. *Phsp-16.2::GFP* worms were aged to day 1, day 6 and day 10 of adulthood, exposed to a 2 hour heat stress at 35°C and then imaged after 4 hours. In each case, we observed a significant increase in reporter activity indicating that the HSR is still able to respond to stress in aged worms.

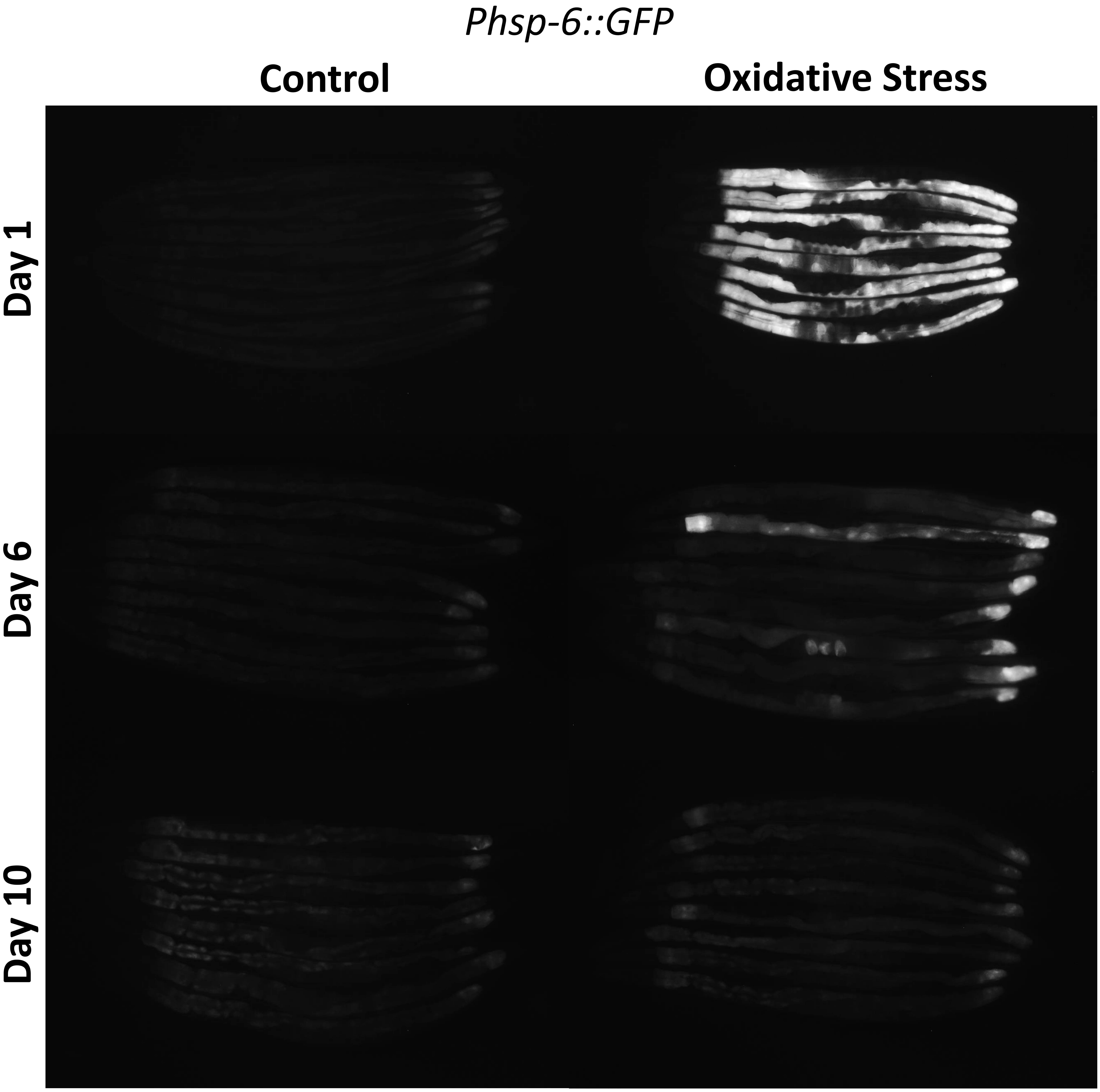

Supplemental Figure S10. Ability to activate mitochondrial unfolded protein response (mito-UPR) is lost with age. *Phsp-6::GFP* worms were aged to day 1, day 4 and day 8 of adulthood, exposed to 4 mM paraquat for 2 days and then imaged. We observed a marked activation of the mito-UPR on day 1 of adulthood. This activation was diminished on day 4 and absent at day 8.

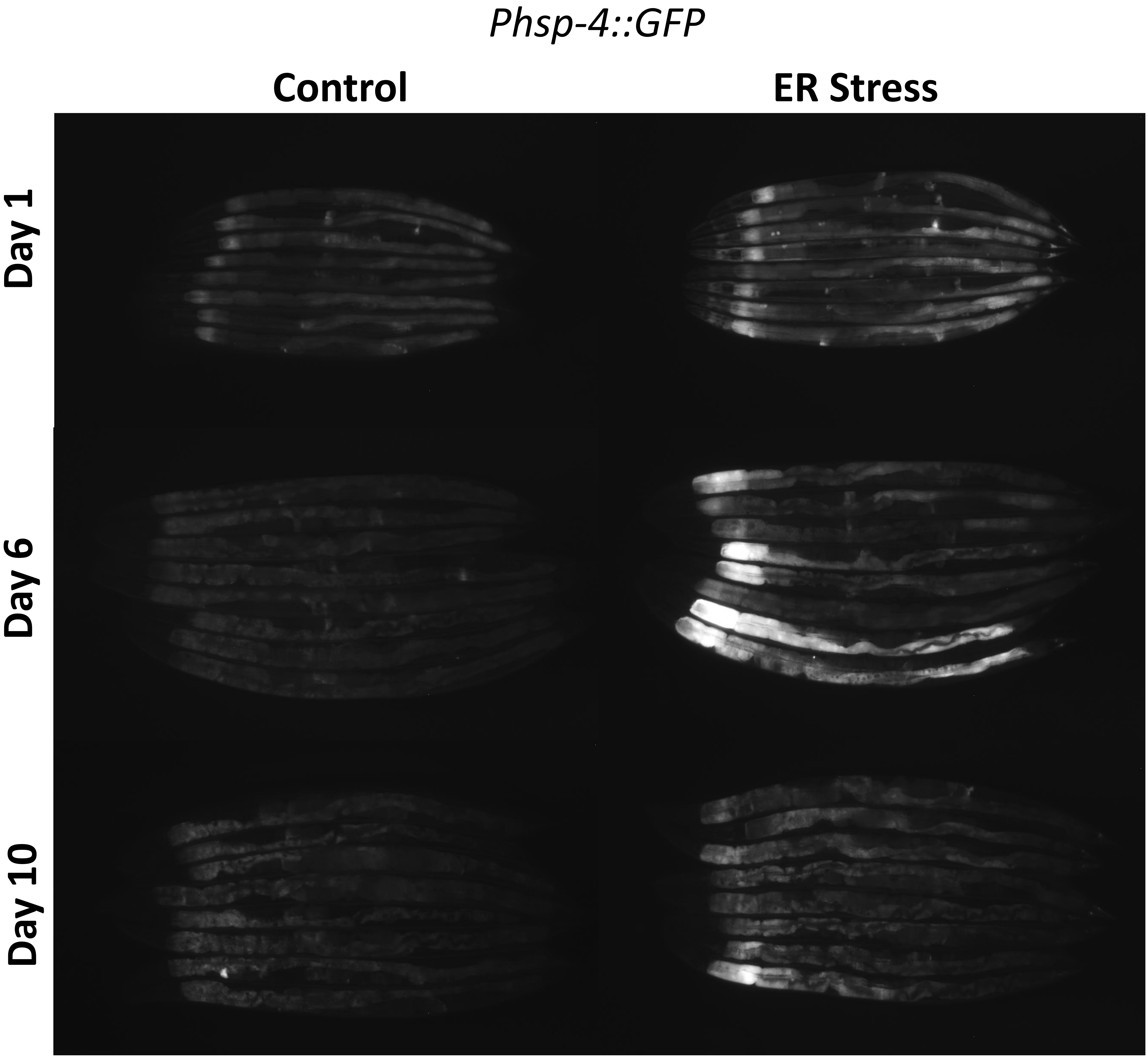

Supplemental Figure S11. Ability to activate endoplasmic reticulum unfolded protein response (ER-UPR) is maintained with age. *Phsp-4::GFP* worms were aged to day 1, day 5 and day 9 of adulthood, exposed to 5 ug/ml tunicamycin for 1 day and then imaged. We observed activation of the ER-UPR at each time point tested.

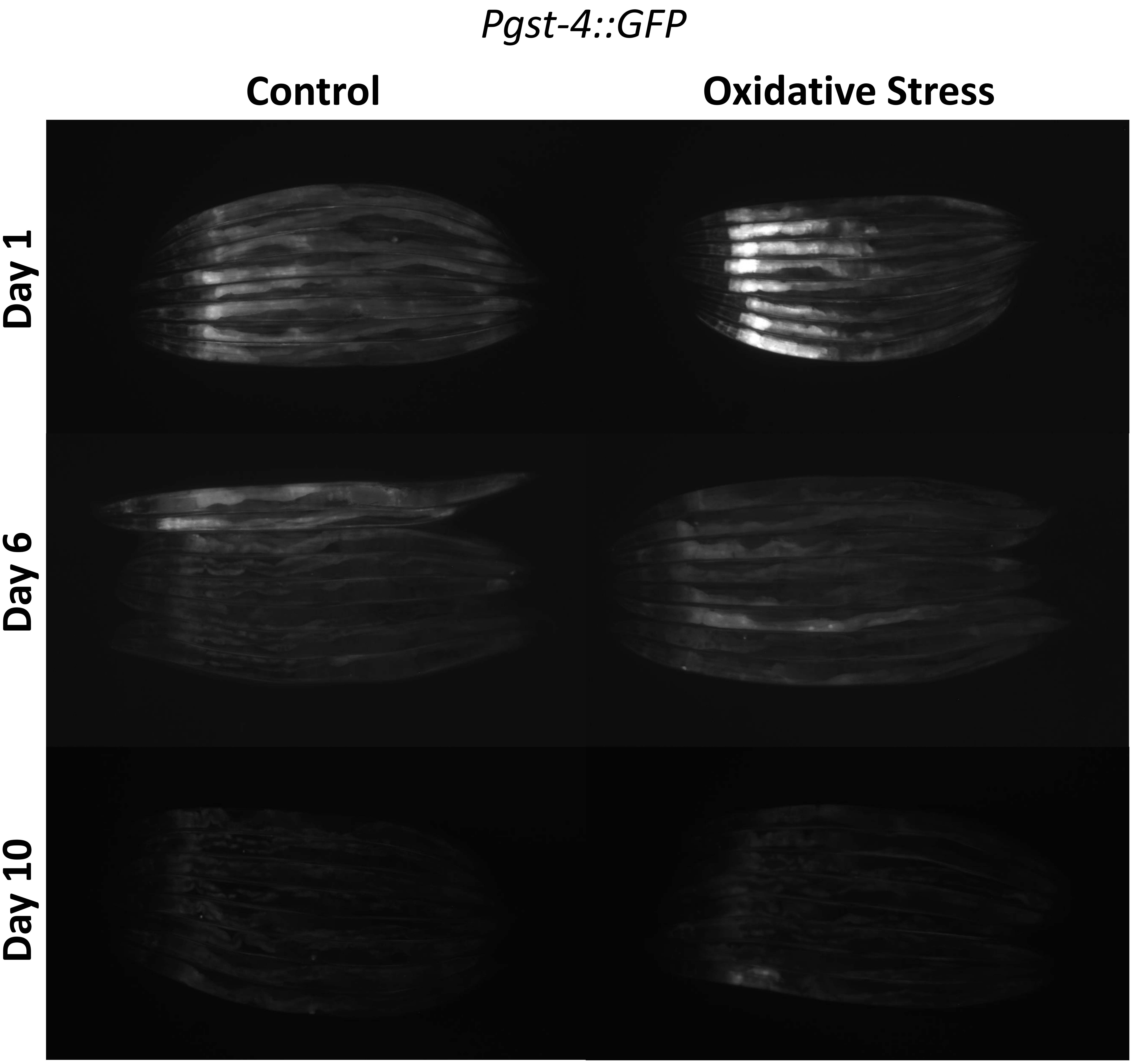

Supplemental Figure S12. Oxidative stress-induced activation of *Pgst-4::GFP* reporter is lost with age. *Pgst-4::GFP* worms were aged to day 1, day 4 and day 8 of adulthood, exposed to 4 mM paraquat for 2 days and then imaged. We observed activation of the *skn-1* mediated detoxification response at day 1 of adulthood but this response was no longer present by day 4.

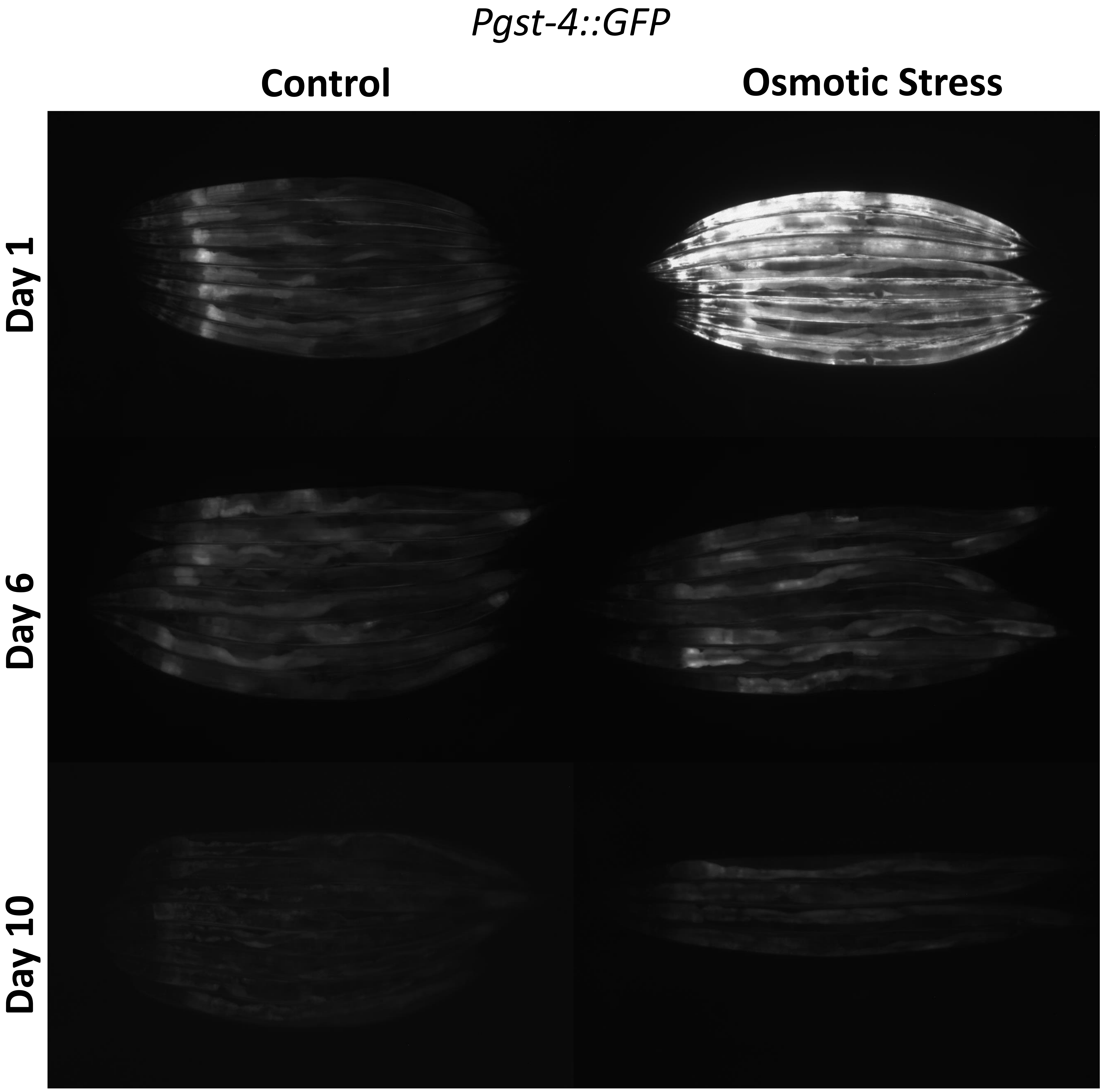

Supplemental Figure S13. Osmotic stress-induced activation of *Pgst-4::GFP* reporter is lost with age. *Pgst-4::GFP* worms were aged to day 1, day 5 and day 9 of adulthood, exposed to 300 mM NaCl for 1 day and then imaged. We observed strong activation of the *skn-1* mediated detoxification response at day 1 of adulthood but this response was no longer present by day 4.

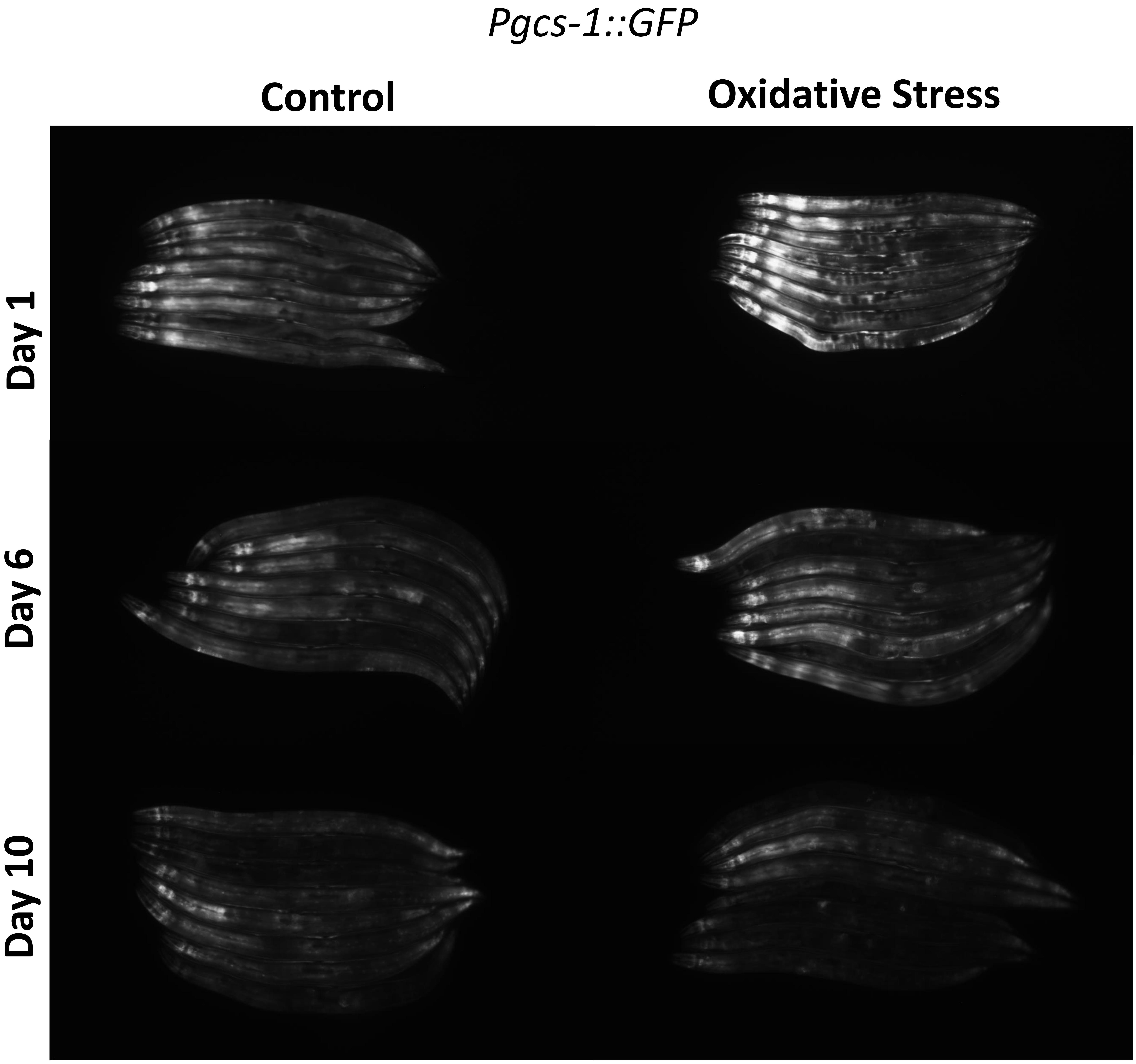

Supplemental Figure S14. Oxidative stress-induced activation of *Pgcs-1::GFP* reporter is lost with age. *Pgcs-1::GFP* worms were aged to day 1, day 4 and day 8 of adulthood, exposed to 4 mM paraquat for 2 days and then imaged. We observed activation of the *skn-1* mediated detoxification response at day 1 of adulthood but this response was no longer present by day 4.

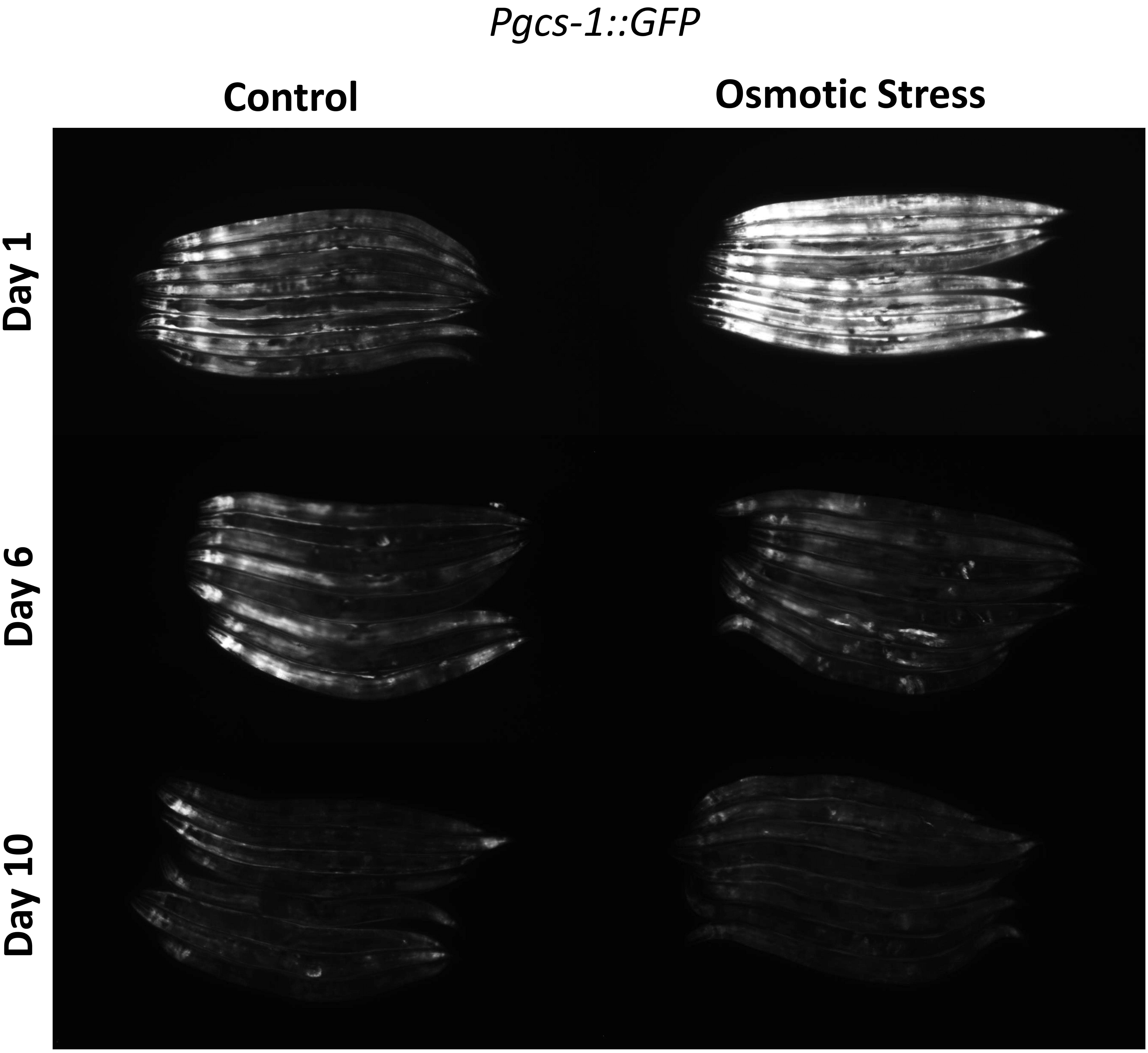

Supplemental Figure S15. Osmotic stress-induced activation of *Pgcs-1::GFP* reporter is lost with age. *Pgcs-1::GFP* worms were aged to day 1, day 5 and day 9 of adulthood, exposed to 300 mM NaCl for 1 day and then imaged. We observed strong activation of the *skn-1* mediated detoxification response at day 1 of adulthood but this response was no longer present by day 4.

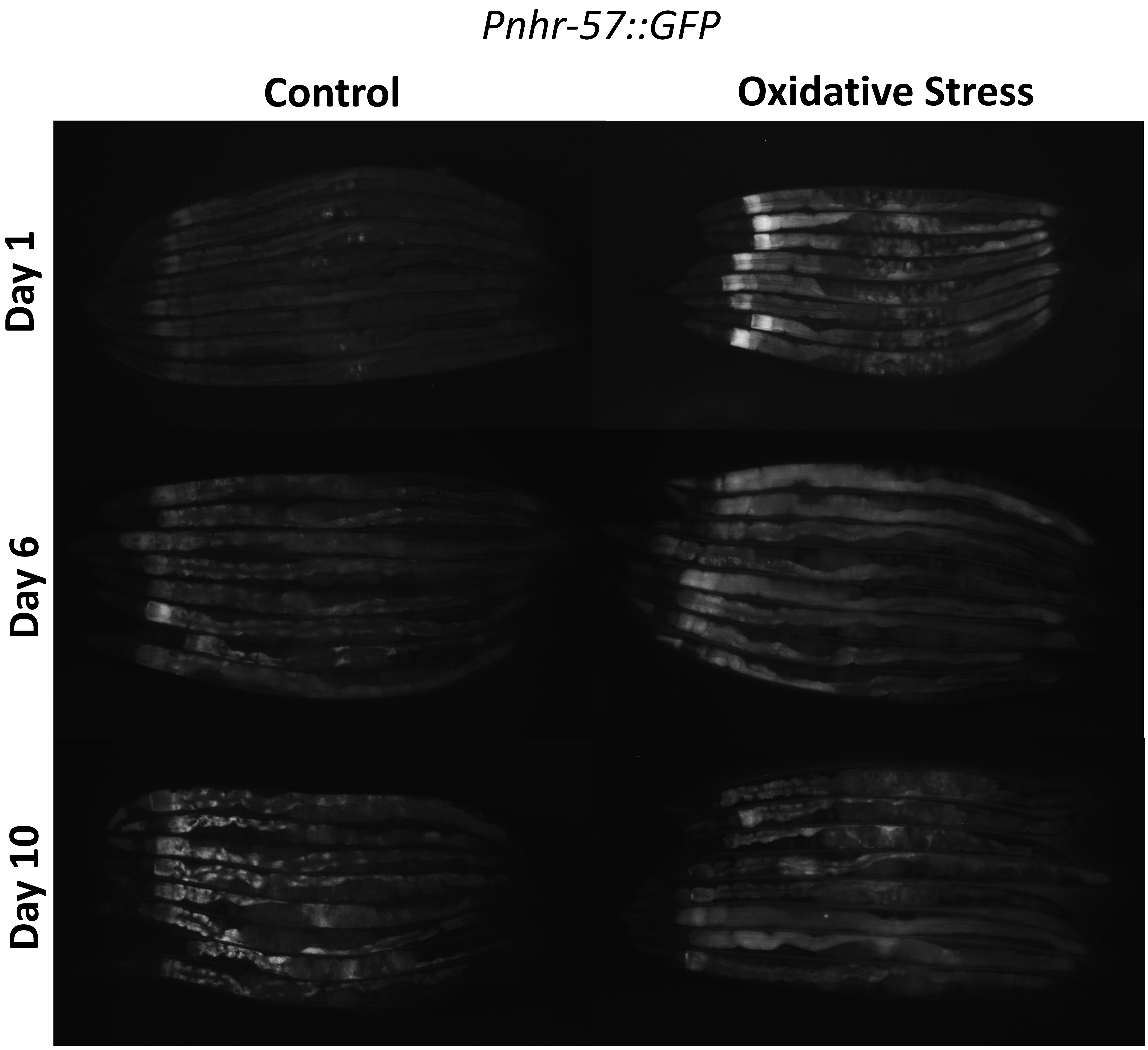

Supplemental Figure S16. Ability to activate hypoxia response decreases with age. *Pnhr-57::GFP* worms were aged to day 1, day 4 and day 8 of adulthood, exposed to 4 mM paraquat for 2 days and then imaged. We observed activation of the hypoxia response at each time point tested but the magnitude of the activation was less at older ages.

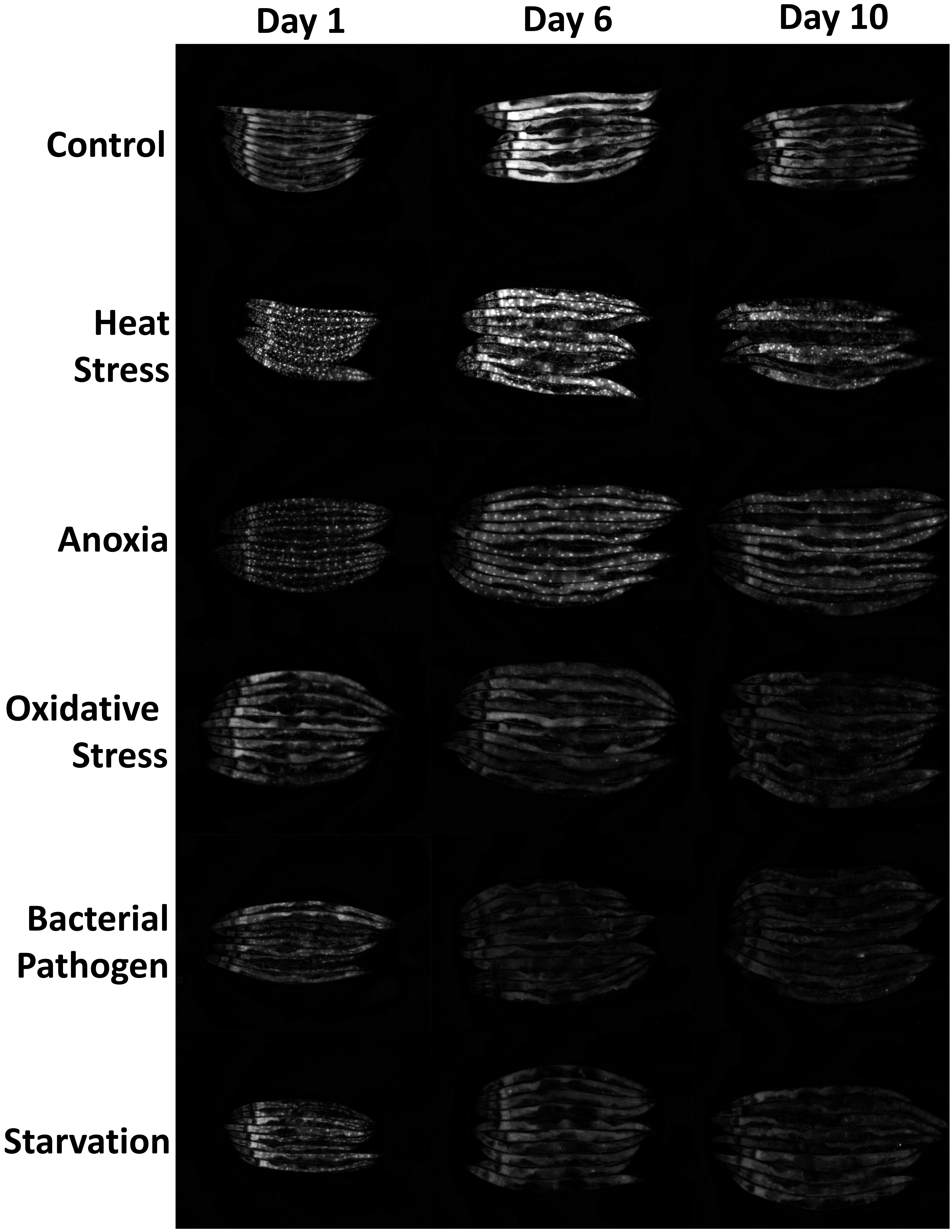

Supplemental Figure S17. Nuclear localization of DAF-16:GFP after stress. Aging *Pdaf-16::daf-16:GFP* worms were exposed to different stresses (35⁰C heat stress for 2 hours, anoxia for 24 hours, 4 mM paraquat for 2 days, *Pseudomonas aeruginosa* for 9 hours, starvation for 48 hours) and imaged. We observed strong nuclear localization of DAF-16:GFP in response to heat stress and anoxia at each age tested. We observed a partial nuclear localization of DAF-16:GFP in response to oxidative stress at all time points. In contrast, we only observed nuclear localization of DAF-16:GFP in response to bacterial pathogens and starvation on day 1 of adulthood.

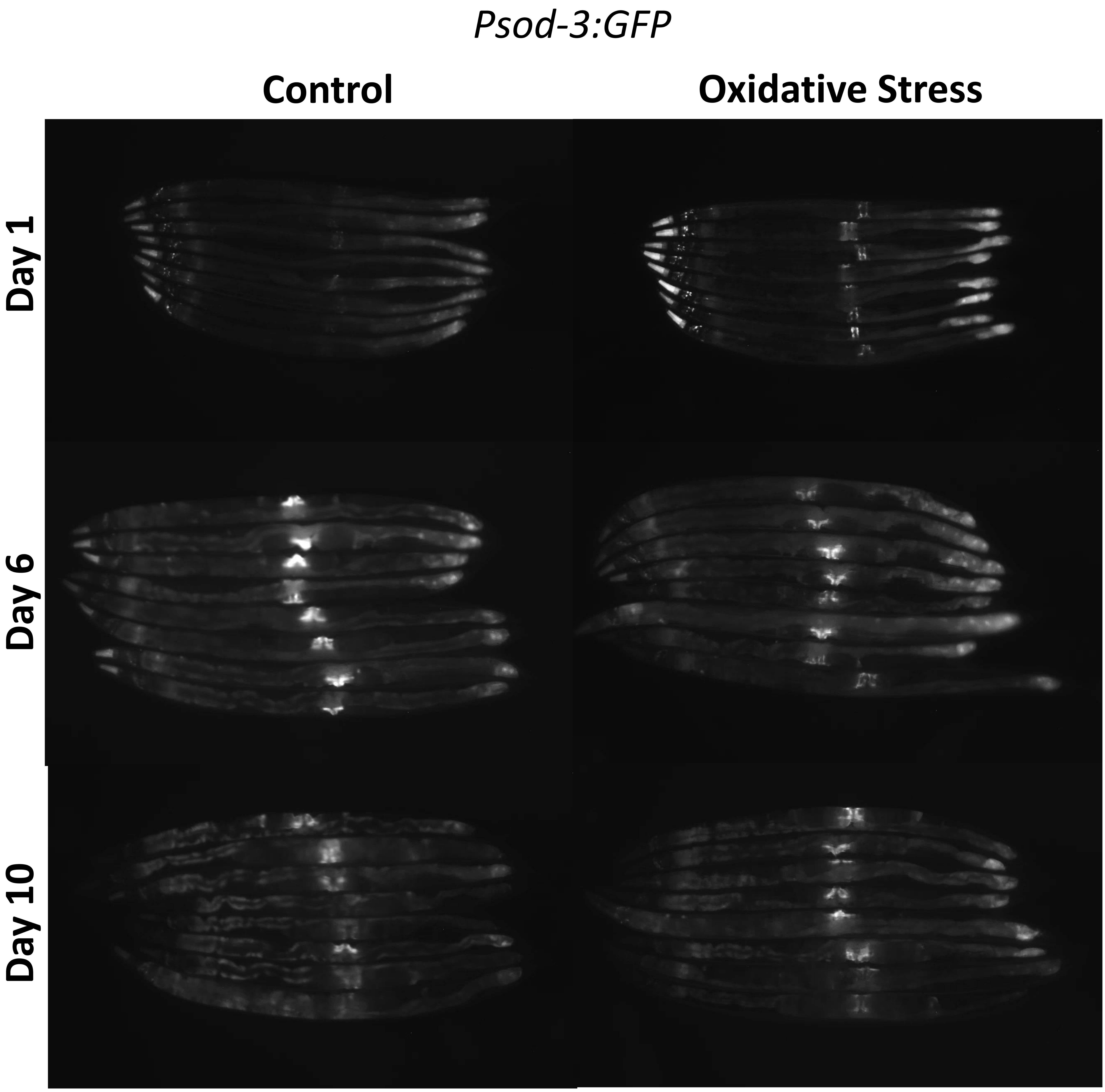

Supplemental Figure S18. Oxidative stress-induced activation of *Psod-3::GFP* reporter is lost with age. *Psod-3::GFP* worms were aged to day 1, day 4 and day 8 of adulthood, exposed to 4 mM paraquat for 2 days and then imaged. While oxidative stress induced the activation of the *Psod-3::GFP* reporter in young worms, this response was lost by day 4 of adulthood.

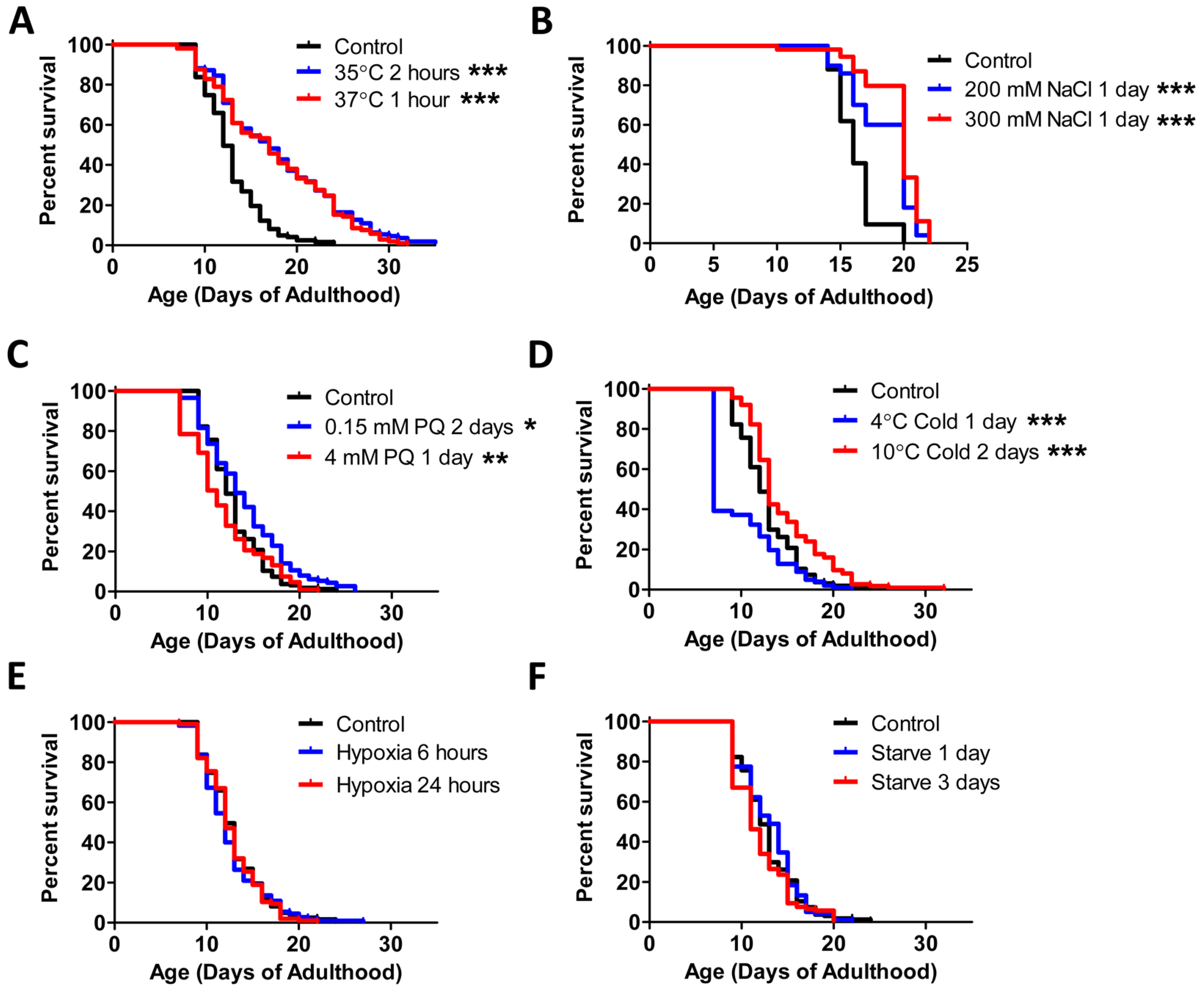

Supplemental Figure S19. Mild exposure to different stresses can cause increase lifespan. Wild-type worms were exposed to mild doses of different types of stress. Under the conditions tested, heat stress (**A**), osmotic stress (**B**), oxidative stress (**C**), and cold stress (**D**) all lead to increases in lifespan to varying degrees. In contrast, exposure to hypoxia (**E**) or short periods of starvation (**F**) did not increase longevity. *p<0.05, **p<0.01, ***p<0.001.

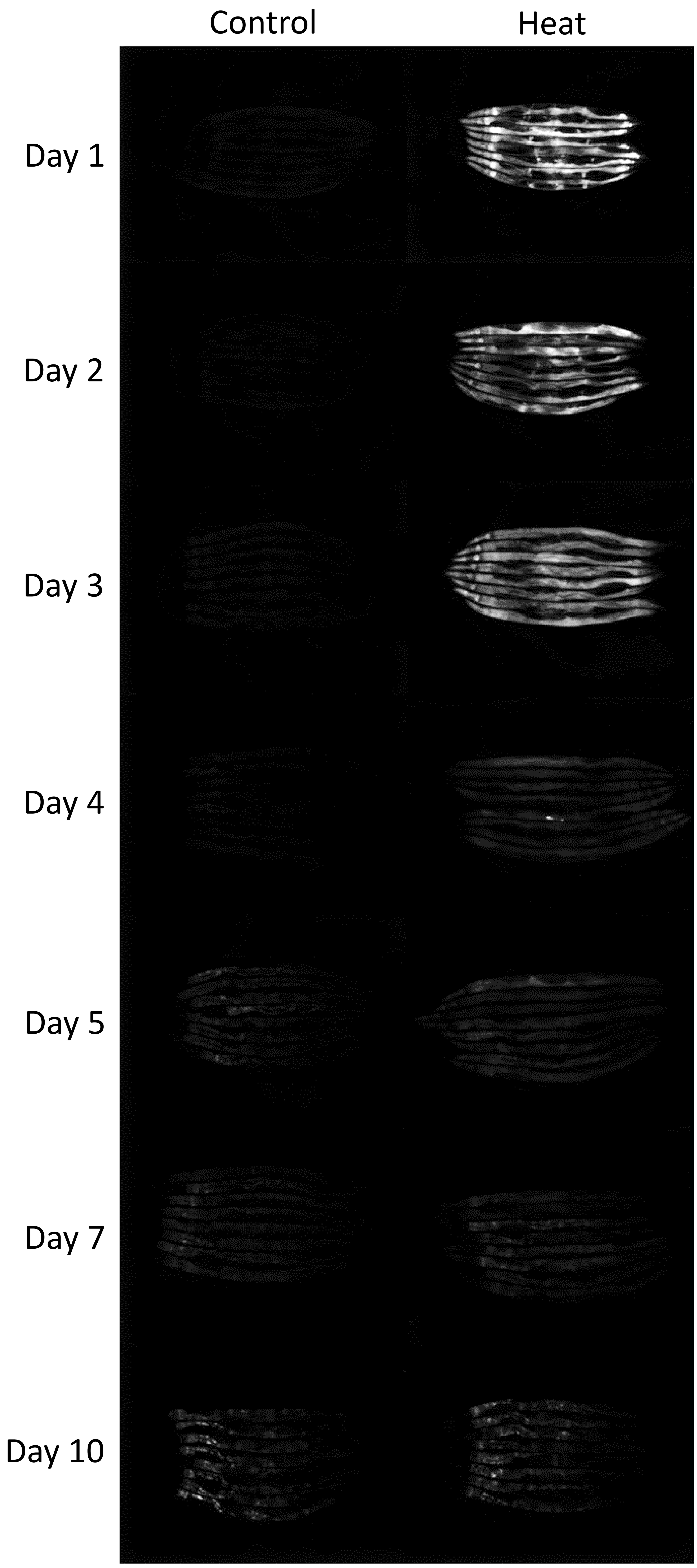

Supplemental Figure S20. Heat shock response (HSR) remains active for multiple days after heat stress. To determine how long the HSR persists after an acute heat stress, we exposed day 1 adult *Phsp-16.2::GFP* worms to 35°C heat for 2 hours and imaged worms daily thereafter. Reporter activity remained highly induced for 3 days and was still significantly increased 5 days later. By 7 days, GFP expression from the *Phsp-16.2* reporter was equivalent between heat-treated and control.

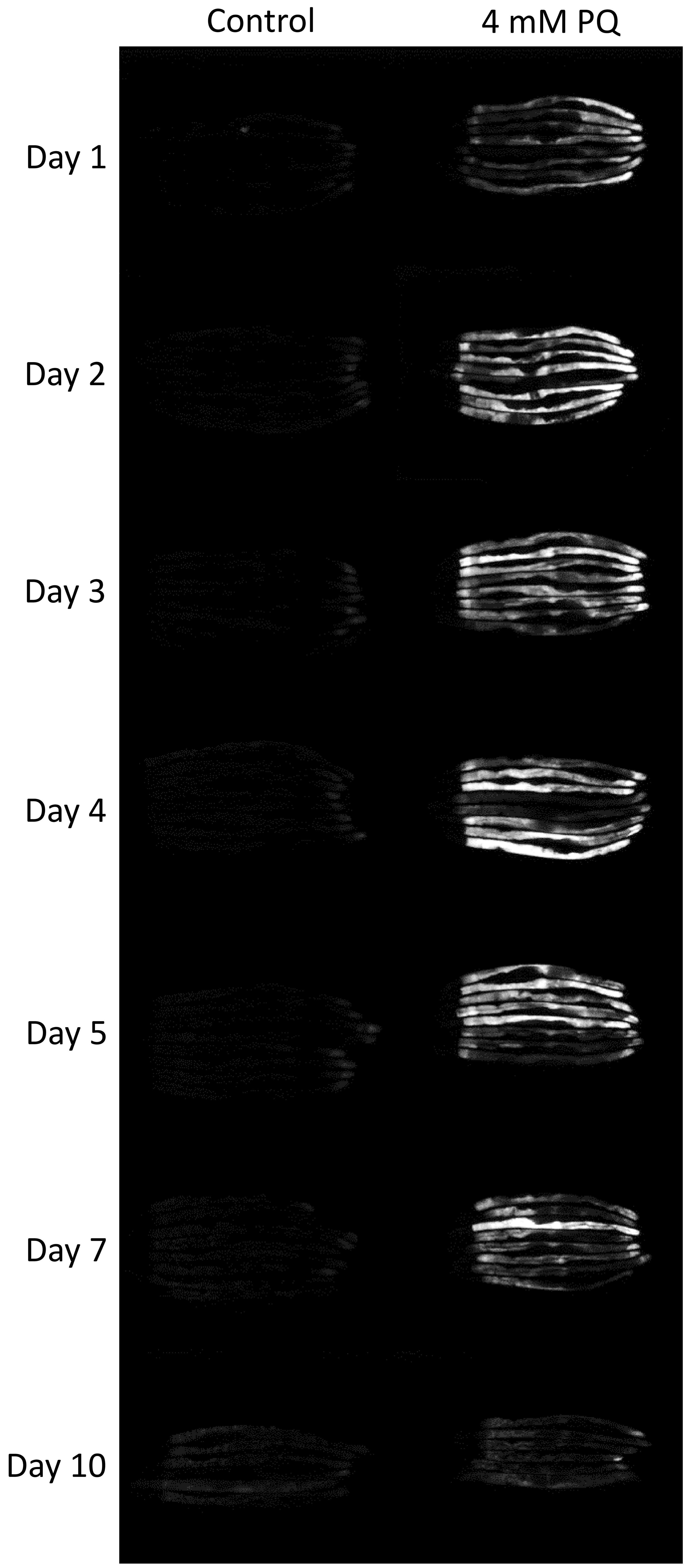

Supplemental Figure S21. Mitochondrial unfolded protein response (mito-UPR) remains active for multiple days after heat stress. To determine how long the mito-UPR persists after an acute oxidative stress, we exposed day 1 adult *Phsp-6::GFP* worms to 4 mM paraquat for 1 day and imaged worms daily thereafter. Reporter activity remained highly induced for 5 days and was still significantly increased 7 days later. By 10 days, GFP expression from the *Phsp-6* reporter was equivalent between oxidative stress-treated and control.

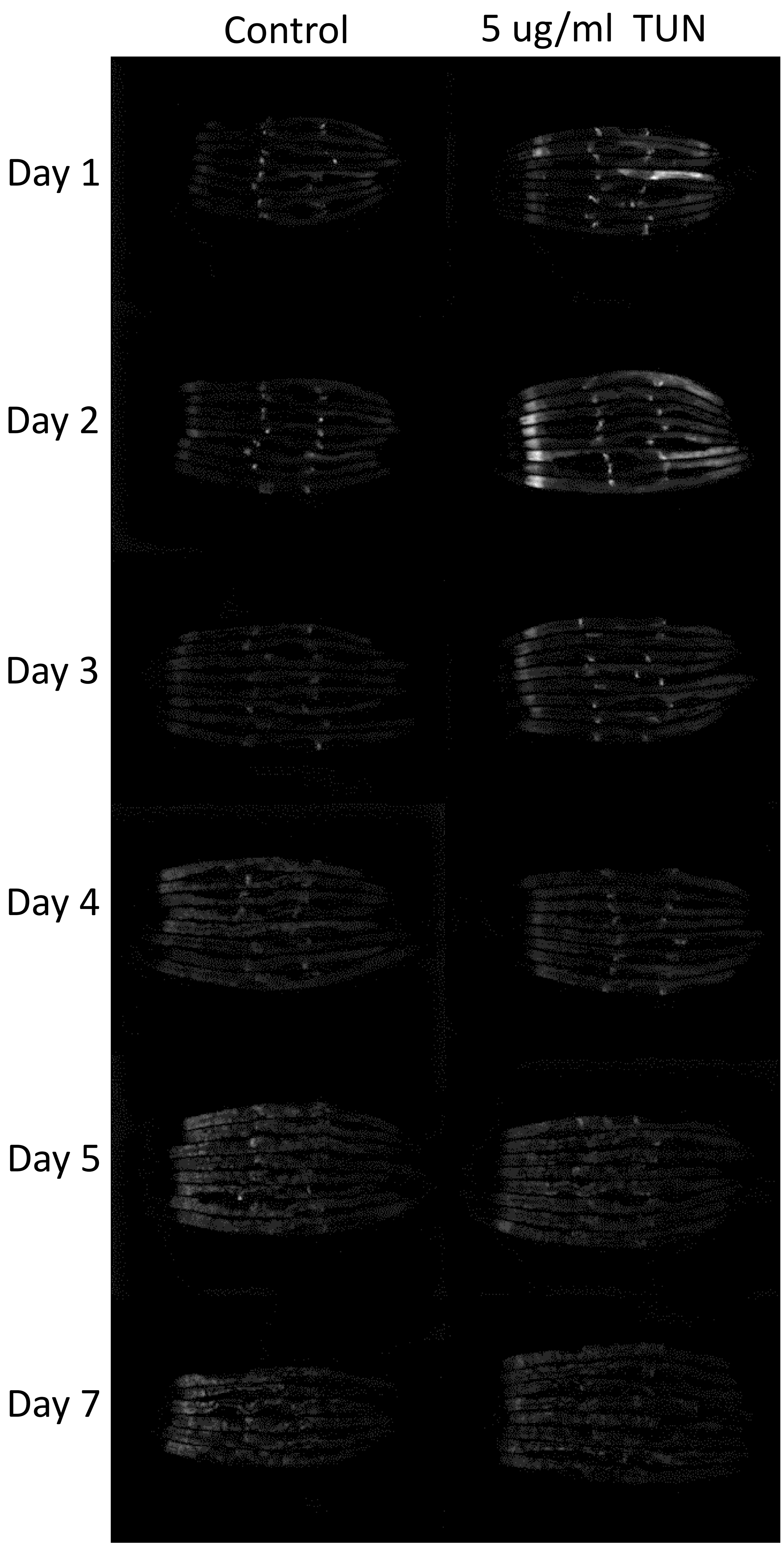

Supplemental Figure S22. Endoplasmic reticulum unfolded protein response (ER-UPR) remains active for multiple days after heat stress. To determine how long the ER-UPR persists after an acute exposure to endoplasmic reticulum stress, we exposed day 1 adult *Phsp-4::GFP* worms to 5 ug/ml tunicamycin for 1 day and imaged worms daily thereafter. Reporter activity was significantly increased for 2 days following ER stress.

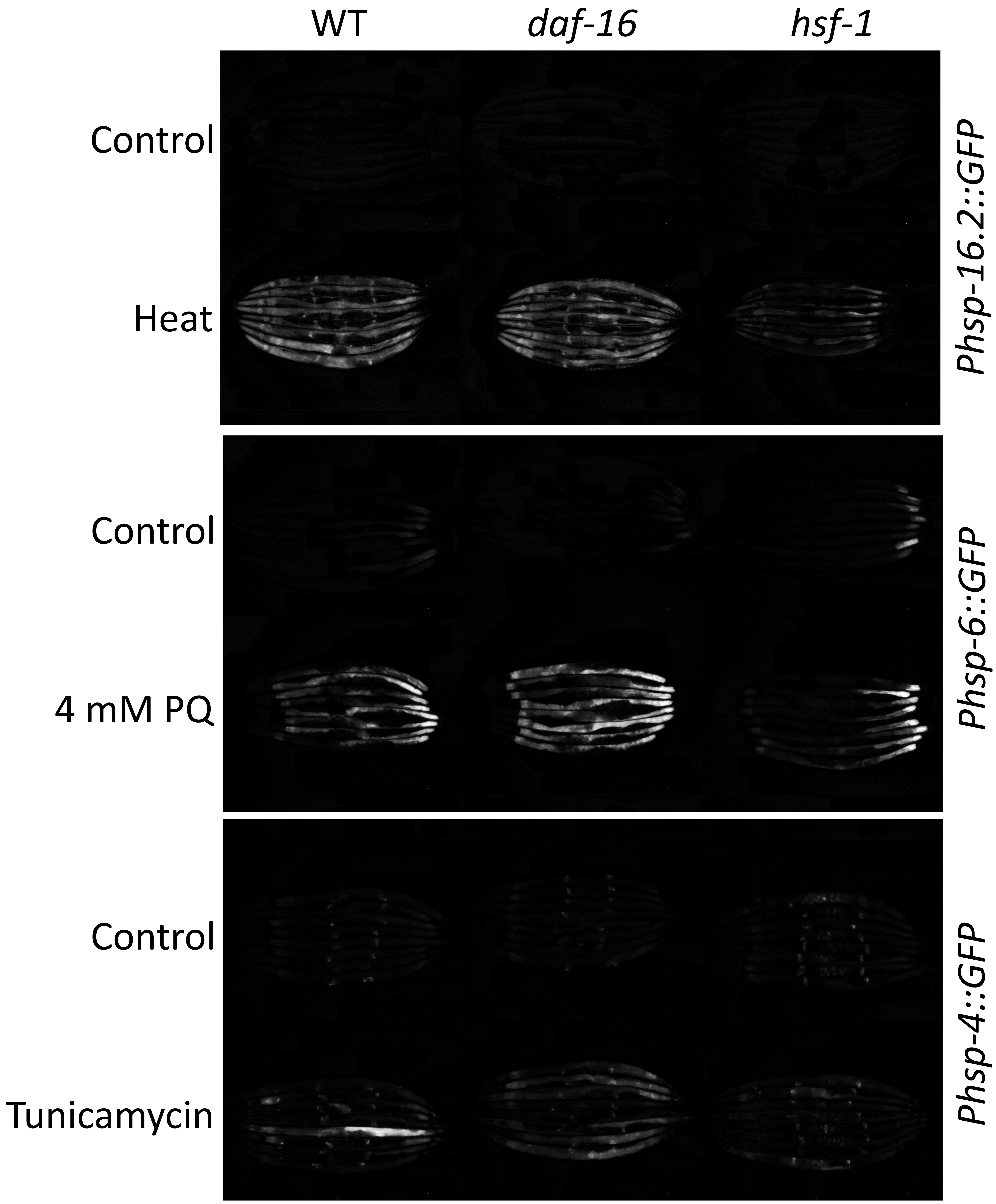

Supplemental Figure S23. HSF-1 is required for full activation of heat shock response, mitochondrial unfolded protein response and endoplasmic reticulum unfolded protein response. Day 1 adult *Phsp-16.2::GFP, Phsp-6::GFP* and *Phsp-4::GFP* animals in a WT, *daf-16* or *hsf-1* background were exposed to 35˚C heat for 2 hours, 4 mM PQ for 1 days or 5 µg/ml tunicamycin for 1 day, respectively. Activation of the *Phsp-16.2::GFP* reporter in response to heat was much reduced in *hsf-1* mutants. Uninduced *Phsp-6::GFP* reporter activity was increased in *hsf-1* worms, while activation in response to oxidative stress was diminished. *hsf-1* mutants also exhibited reduced activation of the *Phsp-4::GFP* reporter in response to ER stress.

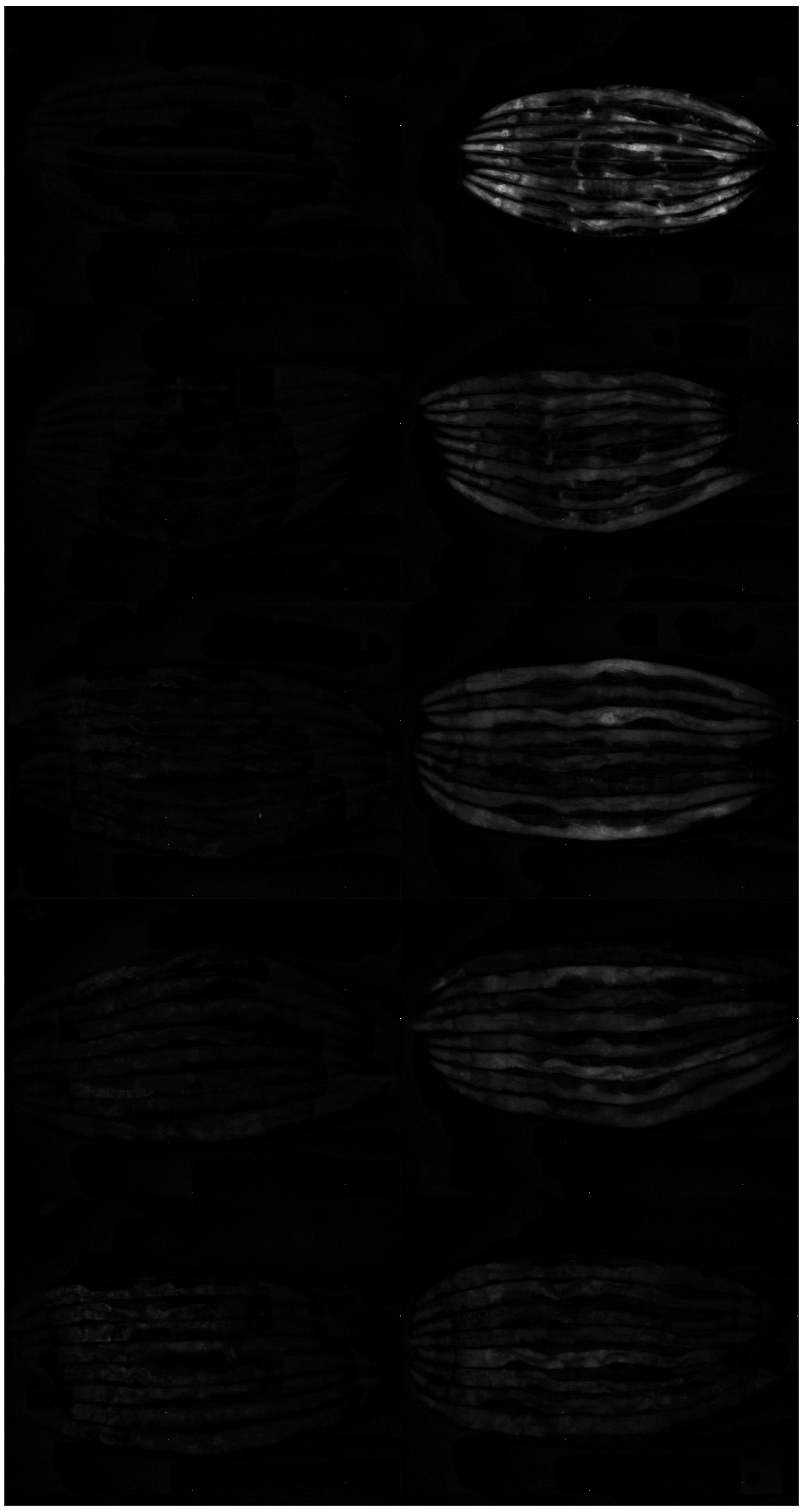

Supplemental Figure S24. Duration of the heat shock response is normal in *daf-16* mutants. Day 1 adult *daf-16;Phsp-16.2::GFP* worms were exposed to 35°C heat for 2 hours and imaged daily thereafter. Reporter activity remained highly induced for 3 days and was still significantly increased 5 days later. As in WT worms, GFP expression from the *Phsp-16.2* reporter was equivalent between heat-treated and control on by day 7 of adulthood.

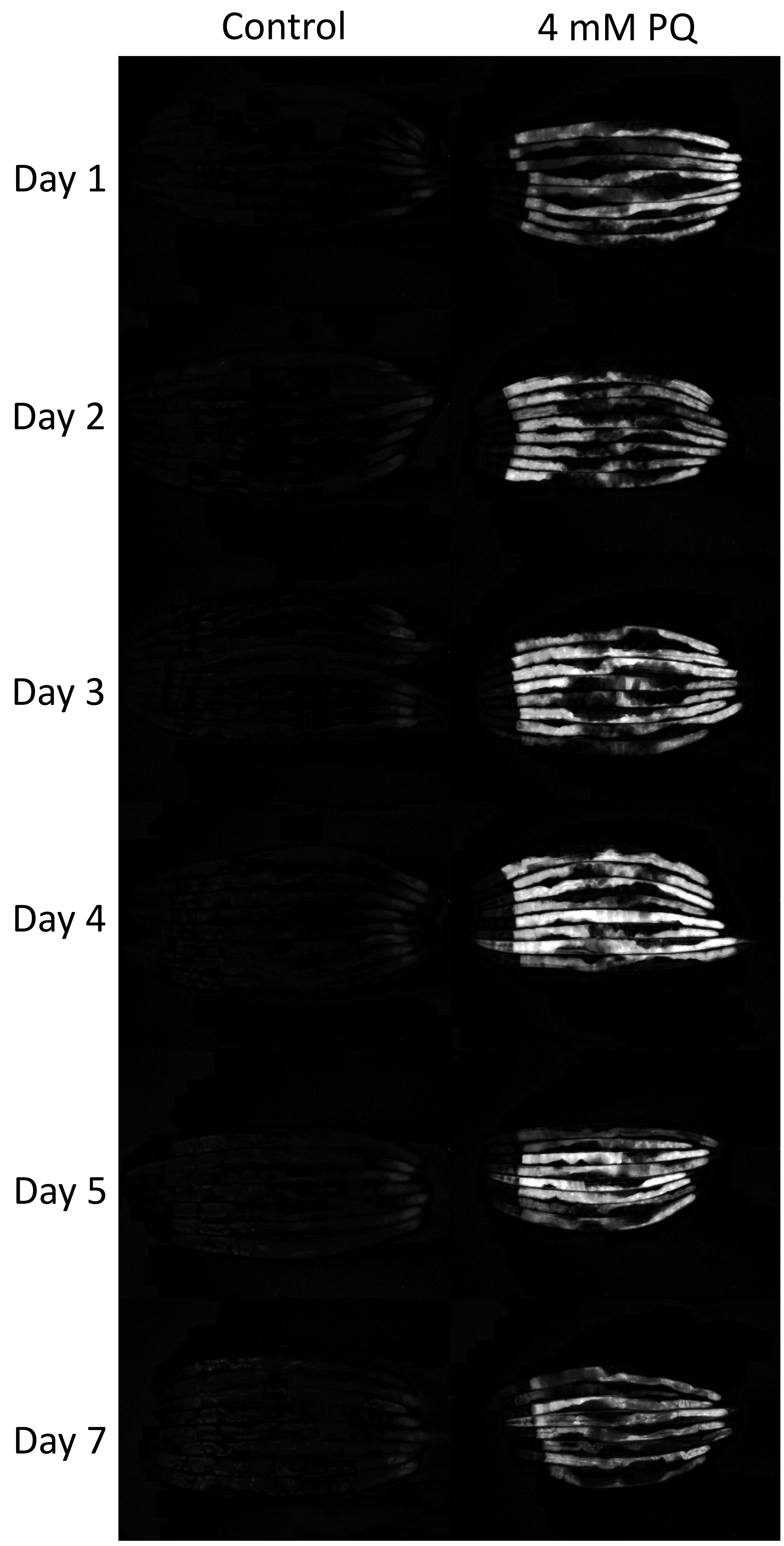

Supplemental Figure S25. Duration of the mitochondrial unfolded protein response (mito-UPR) is normal in *daf-16* mutants. Day 1 adult *daf-16;Phsp-6::GFP* worms were exposed to 4 mM paraquat for 1 day and imaged daily thereafter. As in WT worms, reporter activity remained highly induced for 5 days and was still significantly increased 7 days later.

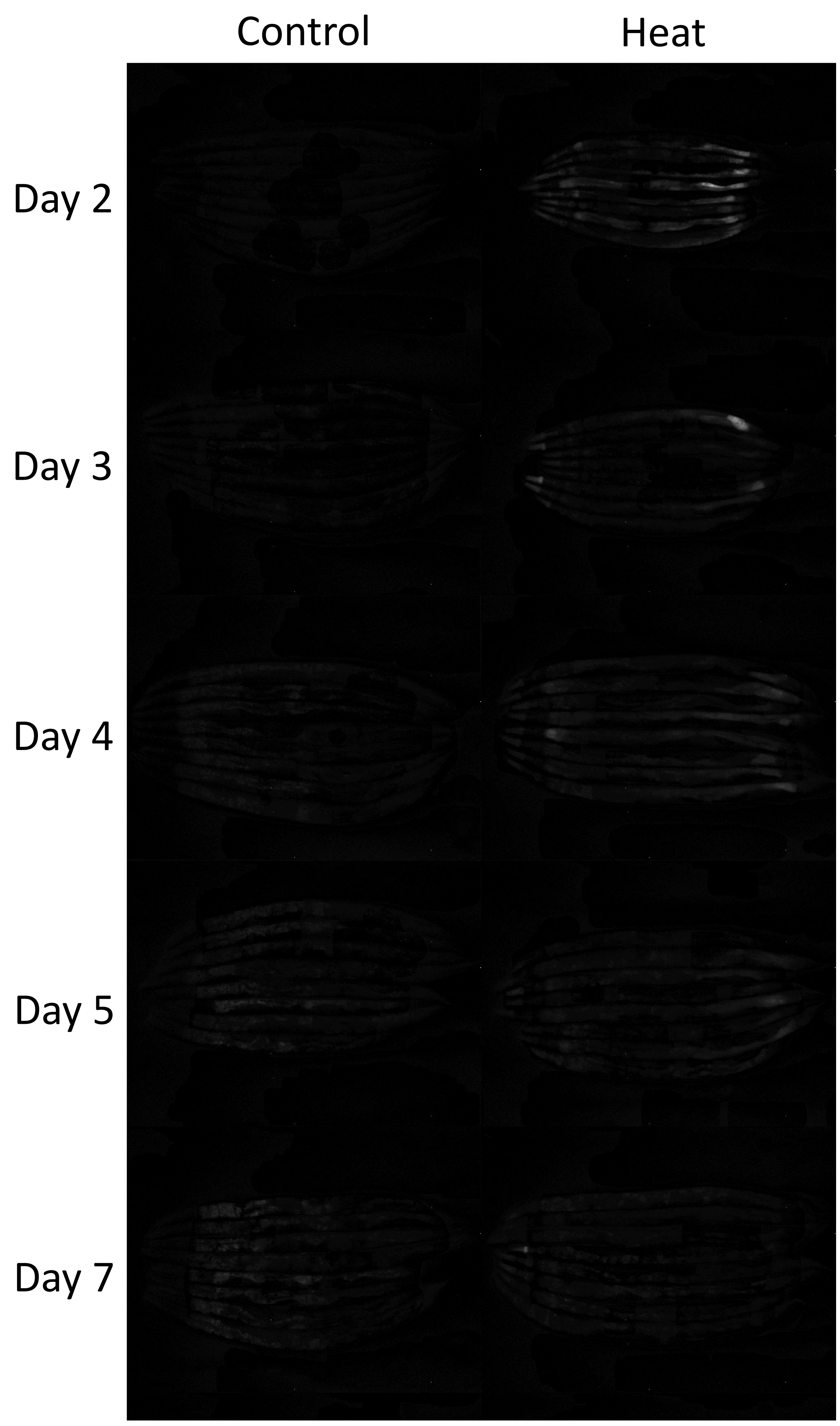

Supplemental Figure S26. Heat shock response is markedly diminished in *hsf-1* mutants. Day 1 adult *hsf-1;Phsp-16.2::GFP* worms were exposed to 35⁰C heat for 2 hours and imaged worms daily thereafter. We observed very mild activation of the *Phsp-16.2::GFP* reporter in *hsf-1* mutants, which remained upregulated until day 5 of adulthood.

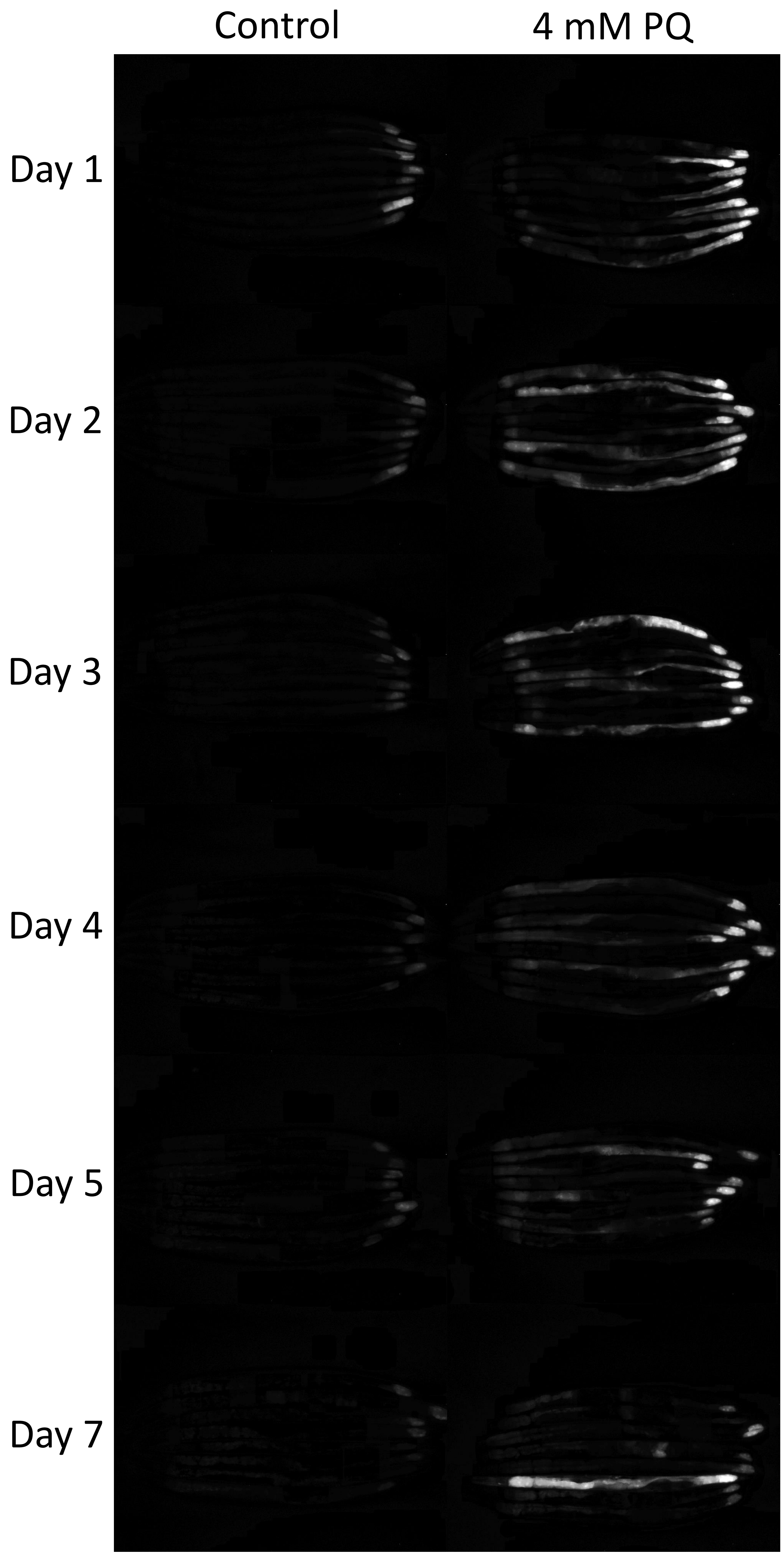

Supplemental Figure S27. Mitochondrial unfolded protein response is diminished in *hsf-1* mutants. Day 1 adult *hsf-1;Phsp-6::GFP* worms were exposed to 4 mM paraquat for 1 day and imaged daily thereafter. In uninduced worms, *Phsp-6::GFP* reporter activity remained higher than WT worms throughout the time course. In contrast, *hsf-1;Phsp-6::GFP* worms show a much milder induction of the mitoUPR when exposed to oxidative stress than WT worms. Nonetheless, the duration of this response was equivalent to WT worms lasting beyond 7 days.

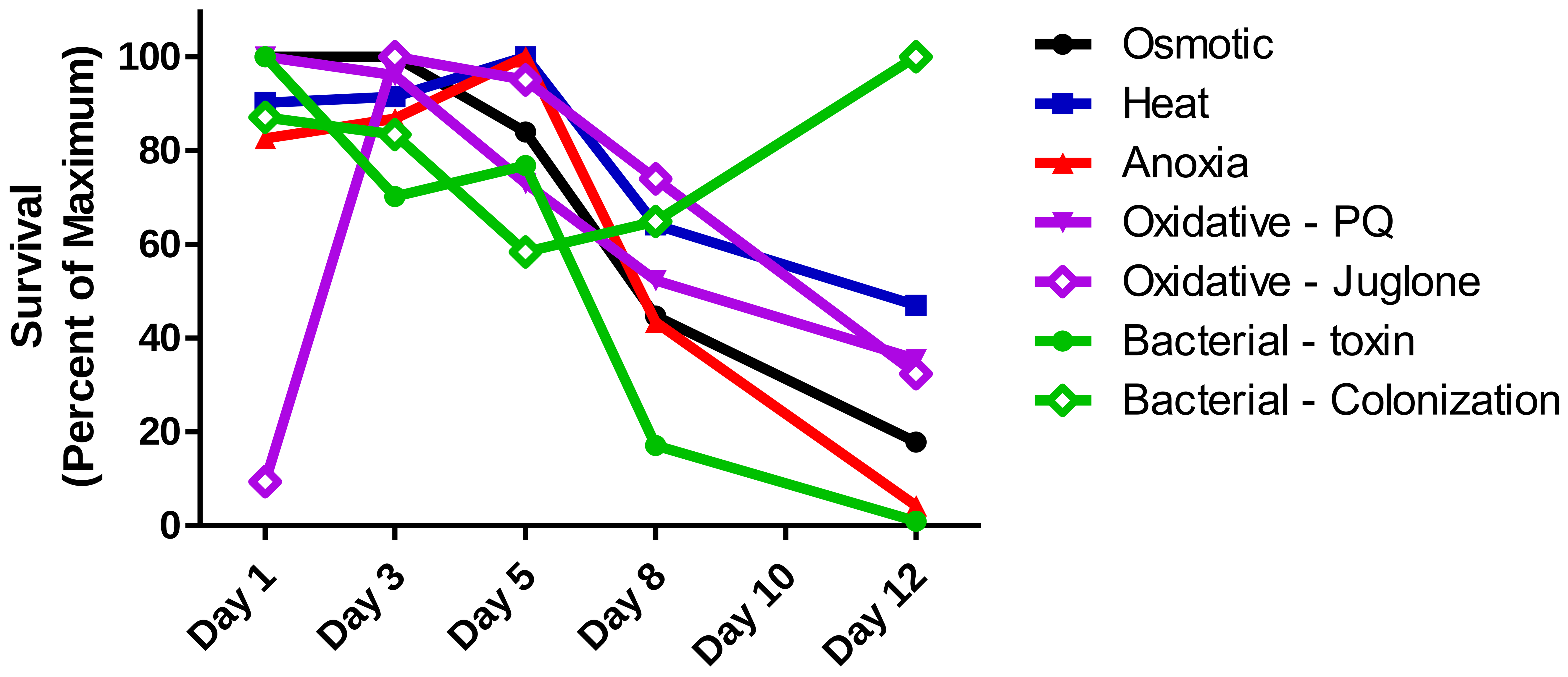

Supplementary Figure S28. Comparison of the decrease of different types of stress with advancing age.

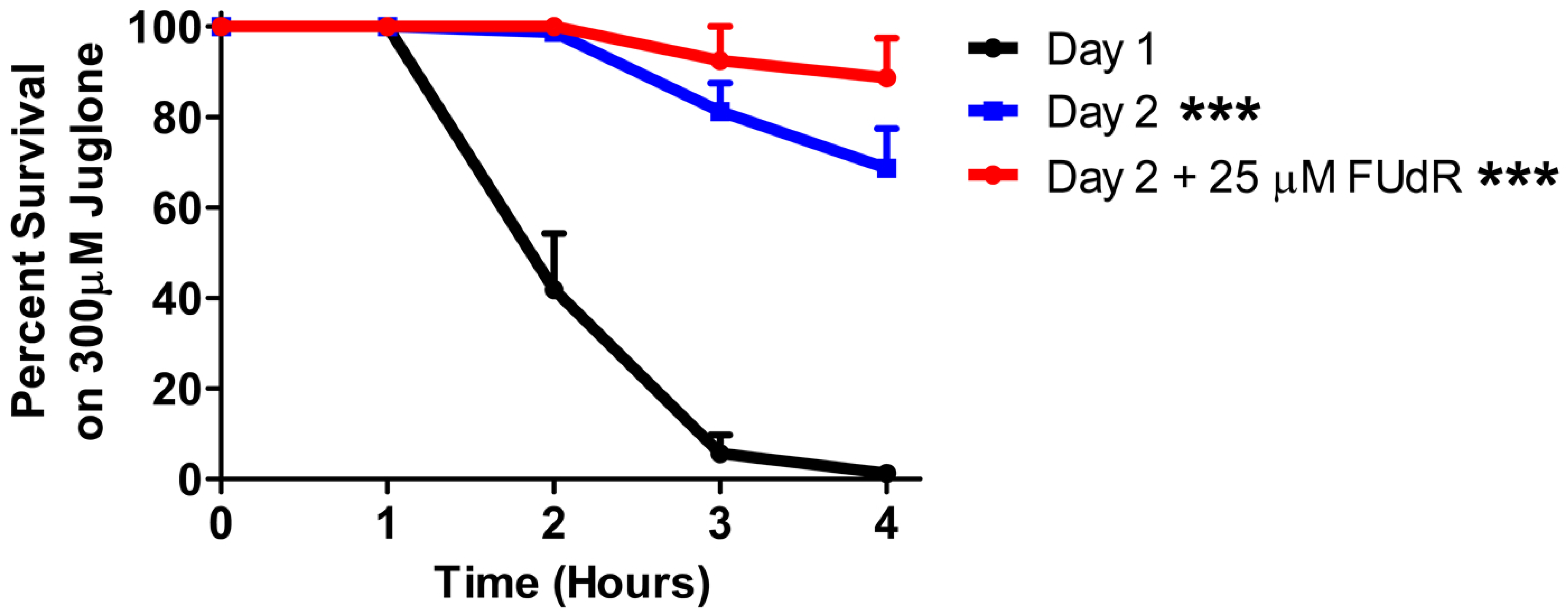

Supplementary Figure S29. Resistance to acute oxidative stress increases after day 1 in absence of FUdR. Day 1 adult worms were transferred to either NGM plates or NGM plates supplemented with 25 µM FUdR. Sensitivity to oxidative stress was tested using the juglone assay on day 2 of adulthood and compared to day 1 adults. Day 2 adults grown on NGM or NGM supplemented with FUdR showed increased resistance to acute oxidative stress compared to Day 1 adults.
